# Trends and projection of incidence, mortality, and disability-adjusted life years of HIV in the Middle East and North Africa (1990–2030)

**DOI:** 10.1038/s41598-023-40743-z

**Published:** 2023-08-24

**Authors:** Zahra Khorrami, Mohammadreza Balooch Hasankhani, Mehrdad Khezri, Ali Jafari-Khounigh, Yones Jahani, Hamid Sharifi

**Affiliations:** 1 Ophthalmic Epidemiology Research Center, Research Institute for Ophthalmology and Vision Science, Shahid Beheshti University of Medical Sciences, No. 23, Paidarfard St., Pasdaran Ave., Tehran, Iran; 2 Modeling in Health Research Center, Institute for Futures Studies in Health, Kerman University of Medical Sciences, Kerman, Iran; 3 Department of Epidemiology, New York University School of Global Public Health, New York, NY United States; 4 HIV/STI Surveillance Research Center, and WHO Collaborating Center for HIV Surveillance, Institute for Futures Studies in Health, Kerman University of Medical Sciences, Kerman, Iran; 5 Road Traffic Injury Research Center, Tabriz University of Medical Sciences, Tabriz, Iran

**Keywords:** Infectious diseases, HIV infections, Statistics

## Abstract

Evidence shows a growing trend of the HIV epidemic in the Middle East and North Africa (MENA). We aimed to project the incidence, mortality, and disability-adjusted life years (DALY) in the region from 1990 to 2019 and assess its trend by 2025, and 2030. We extracted the HIV incidence, mortality, and DALY data from the Global Burden of Disease (GBD) and UNAIDS databases. The joinpoint regression model was used to examine changes in HIV trends. The trend changes were estimated by average annual percent change (AAPC). In most countries, an increasing trend was observed in HIV incidence, mortality, and DALY. Specifically, the highest growth in the annual incidence rate was related to Egypt (AAPC = 14.4, GBD) and Iran (AAPC = 9.6, UNAIDS). Notably, Qatar (AAPC = − 5.6, GBD), Bahrain (AAPC = − 3.3, GBD), and Somalia (AAPC = − 4.2, UNAIDS) demonstrated a significant reduction in incidence. Regarding mortality rates, Djibouti (AAPC = 24.2, GBD) and Iran (AAPC = 16.2, UNAIDS) exhibited a significant increasing pattern. Furthermore, the estimated increase in incidence by 2030 was most marked in Djibouti (985%) and Iran (174%). Iran (422%) and Egypt (339%) showed a prominent rise in mortality rates. GBD data showed 16 countries had an increasing pattern in DALY in both genders. According to age and period effects, there was a significant upward trend in incidence, mortality rates, and DALY. Findings highlighted the urgent need for improved prevention and treatment services, including expanding access to HIV testing, promoting safe practices, increasing antiretroviral therapy coverage, and supporting targeted interventions for high-risk populations.

## Introduction

Worldwide, HIV continues to be a significant public health concern and has destructive health outcomes^1^. Reports globally show that in 2020, 37.7 million people were living with HIV, 1.5 million people became newly infected with HIV, and 680,000 people died due to AIDS-related consequences^2^. With the promising effectiveness of antiretroviral therapy (ART) to treat the infection and prevent transmission and worldwide efforts to implement treatment and prevention programs, UNAIDS declared the intention to end AIDS as a public health threat by 2030. In this regard, UNAIDS developed strategies for enhancing HIV response among societies to achieve 90% coverage for HIV testing, treatment, and sustained viral suppression by 2020 and 95% coverage by 2030^3^. However, the burden of the epidemic continues to vary substantially between countries and regions, and there remains limited information about trends and projections of the incidence and mortality of HIV in some regions.

There are some barriers to the control of HIV in the Middle East and North Africa (MENA) including stigma and discrimination around key populations, internal wars, terrorism, and political problems^4^. These barriers lead to the region being one of the only world regions with rapidly rising HIV cases, making it a substantial area of concern within the global HIV landscape^5^. For example, this region, with 43% antiretroviral therapy coverage, has the lowest antiretroviral therapy coverage across all world regions^6^. Moreover, the HIV epidemic in some countries of the region among HIV key populations, including female sex workers (FSWs), People who inject drugs (PWID), and men who have sex with men (MSM) is concentrated^5^. While HIV epidemics have emerged over the past 20 years among these key populations in the region, evidence suggests that HIV incidence appears to have increased in the region since 2010^7^. For example, a recent modelling study estimated that the number of new HIV infections in 2020 in the MENA was 3471 in FSWs, 6416 in their clients, and 4717 in the client spouses^7^. Another study estimated the HIV incidence rate in 2017 among PWID overall ranges widely from 0.7% per person-year in Tunisia and 7.8% per person-year in Pakistan to as high as 24.8% per person-year in Libya^8^. Among MSM, HIV prevalence was found to vary from 3.6% in Lebanon to 9.3% in Sudan, and the highest measured prevalence was 13.0% in Tunisia^9^. Despite the rapidly rising HIV epidemic and the fact that the HIV response in the MENA remains below global targets for prevention, testing, and treatment, the status of HIV incidence and mortality and their trends remains unknown. Notably, no model assessed the distribution trends of incidence, mortality, and disability-adjusted life years (DALY) of HIV in the region.

Utilizing joinpoint regression analysis can provide crucial insights into the dynamic changes over time, identify significant shifts in trends, and shed light on the evolving HIV epidemic in the MENA region. The joinpoint model does not have requirements for the distribution of data and can describe the long-term trend in a linear model. The basic idea is to divide a long-term linear trend into several segments, each of which is described by a continuous linear pattern^10^. However, to the best of our knowledge, no previous studies have comprehensively evaluated and quantified the trend of HIV incidence, mortality rates, and DALY within the MENA region. To address this evidence gap and consider the escalating HIV epidemic in the MENA region, this study aimed to analyze the trend changes and region distribution changes of HIV incidence and mortality between 1990 and 2019 using a joinpoint regression analysis. Additionally, the study sought to estimate the changes in incidence, mortality, and DALY by 2025 and 2030 in the MENA region. The findings of this study can inform strategies for enhancing access to HIV prevention and treatment services in the MENA. Moreover, the findings can provide a scientific basis for the governments to improve HIV prevention and control measures in the region.

## Methods

### Study design and data collection

In this ecological study, two sources of data from 21 countries, including Algeria, Bahrain, Djibouti, Egypt, Iran, Iraq, Jordan, Kuwait, Lebanon, Libya, Morocco, Oman, Palestine, Qatar, Saudi Arabia, Somalia, Sudan, Syria, Tunisia, United Arab Emirates (UAE), and Yemen were used. The first source of data was age-standardized incidence, mortality rates (ASRs), and disability-Adjusted Life Years (DALY) of HIV in 21 countries from the Global Burden of Disease (GBD) site, owned by the Institute for Health Metrics and Evaluation (IHME), University of Washington^11^. The second source of data was the data reported in 14 countries by UNAIDS, which was extracted from the official UNAIDS website^12^.

GBD provides comprehensive data on various health indicators, including incidence, prevalence, death (Years of life lost: YLLs), Years Lived with Disability (YLDs), and DALY for each disease and injury in each year, location, age group, and sex estimates and reports for 204 countries from 1990 to 2019. UNAIDS maintains a nationwide database of AIDS information reports, including estimates of HIV prevalence, incidence, mortality, and DALY associated with AIDS. The majority of these estimates are generated using Spectrum software^13^.

Informed consent was not applicable. Ethical approval was obtained from the Ethics Committee of Kerman University of Medical Sciences (Ethics Code: IR.KMU.REC.1401.263) and all methods were performed under the relevant guidelines and regulations.

### Statistical analysis

Joinpoint regression was used to analyse the time trend and identify significant changes in the incidence, mortality rate, and DALY of HIV from 1990 to 2019^14^. The joinpoint regression model is a method of segmenting the nonlinear regression model into separate linear fragments in which the fragments are separated by joinpoints. We performed a logarithmic transformation of the ASRs and computed the standard errors adopting binomial approximation. Annual Percent Change (APC) indicate the amount of increase or decrease in the percentage of change per year, which shows a decreasing trend if negative, an increasing trend if positive, and the Average Annual Percent Change (AAPC) is the average all these changes, which are calculated to describe linear trends based on each period.

To determine the number and locations of breakpoints and estimate the regression parameters of the components, Joinpoint regression software version 4.9.0.1 was used, which is specifically designed to perform connection point regression analysis. This software uses 4499 repetitions of the permutation test to estimate the failure points by default, in which the number of failure points was considered to be a maximum of four. The significance level of the tests was considered less than or equal to 0.05.

### Prediction model

We estimated the percent change in incidence, mortality, and DALY by 2025 and 2030 when compared to the final segment (trend 5), and compute the standard errors of the projection, using the betas displayed on the APC in the final segment^15,16^.

### Age–period–cohort analysis

We described the magnitude of the rates as a function of age (a), period (p), and birth cohort (c) using a log-linear model, with Poisson distribution and with the log of the person-years at risk defined as an offset of the method. Age-period-cohort model analyses were conducted using APC fit with the APC package in R, version 3.6.0^17^.

### Sensitivity analysis

The sensitivity analysis was performed in four steps. First, starting with the selection data as below:

Data from 1993 to 2013 were used to predict HIV incidence in 2014.

Data from 1994 to 2014 were used to predict HIV incidence in 2015.

Data from 1995 to 2015 were used to predict HIV incidence in 2016.

Data from 1996 to 2016 were used to predict HIV incidence in 2017.

Data from 1997 to 2017 were used to predict HIV incidence in 2018.

Second, we fitted the Joinpoint Regression model for each of the datasets.

Third, we estimated the prediction in incidence, mortality, and DALY using the betas displayed on the APC in the final segment.

To measure the accuracy of the prediction counts, the prediction error is estimated for each year by computing the predicted minus the observed value. We then find the ratio of the prediction error to the observed count, adding 0.5 to the denominator to avoid numerical error when the observed count is zero. The ratio is the percentage error of the prediction, compared to the observed count:

$$error \,ratio=\frac{Predicted-Observed}{Observed+0.5}.$$

Fourth, the average absolute relative deviation (AARD) was computed as the average of the error ratios over all the cases considered in the data. More specifically, assume $\widehat{{\theta }_{S}}$ is the predicted mortality for specific scenario s, s = 1,…, S, and that ${\theta }_{S}$ is the true observed count.

$$AARD=\frac{1}{S}\sum_{S=1}^{S}\frac{\left|\widehat{{\theta }_{S}}-{\theta }_{S}\right|}{{\theta }_{S}+0.5}.$$

Smaller values in AARD indicate closer estimates to the true values^15,16^.

The results of the prediction model of the incidence rate of Algeria country between 1993 and 2019 were shown in Supplementary Tables S1 and S2.

### Ethics approval

Ethical approval was obtained (IR.KMU.REC.1401.263) from the Ethics Committee of Kerman University of Medical Sciences.

## Results

### Age-standardized incidence rate trend

**Figure 1:**
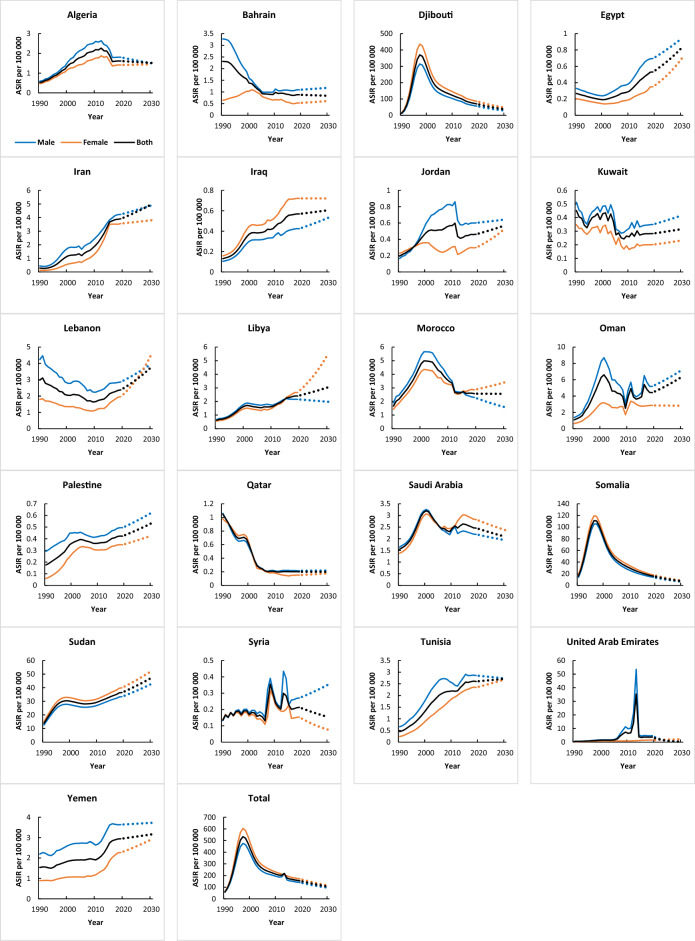
Trends in age-standardized incidence rates (1990–2019) and prediction (2020–2030) per 100,000 from HIV in MENA countries (GBD).

According to the GBD database, out of the 21 countries analyzed, 14 countries had an increase in incidence, two countries showed a declining trend, and five countries reported stable trends from 1990 to 2019. According to the GBD database, Iran had the highest annual growth rate in HIV incidence (AAPC = 9.6, 95% CI 8.8, 10.5; P<0.001), and Morocco had the lowest annual growth rate (AAPC = 1.4, 95% CI 0.6, 2.2; P < 0.001). Qatar (AAPC = − 5.6, 95% CI − 6.1, − 5.0; P < 0.001) and Bahrain (AAPC = − 3.3, 95% CI − 4.3, − 2.3; P < 0.001) had the age-standardized incidence rate between 1990 and 2019 (Table 1 and Fig. 1). Furthermore, the results of modelling with UNAIDS data showed that the incidence of HIV has decreased annually in Somalia (AAPC = − 4.2, 95% CI − 4.9, − 3.5; P < 0.001), Djibouti (AAPC = − 2.0, 95% CI − 3.9, − 0.0; P = 0.049), and Morocco (AAPC = − 0.7, 95% CI − 1.0, − 0.3; P < 0.001). The highest annual growth rate was related to Egypt (AAPC = 14.4, 95% CI = 14.1, 14.8; P < 0.001), and the lowest annual growth rate was related to Lebanon (AAPC = 1.9, 95% CI 1.6, 2.1; P < 0.001) (Table 1).

**Table 1:** Joinpoint regression analysis of age-standardized incidence rates, and incidence rates from HIV in MENA countries, 1990–2019.

Country	Trend 1		Trend 2		Trend 3		Trend 4		Trend 5	
	Years	APC (95% CI)	Years	APC (95% CI)	Years	APC (95% CI)	Years	APC (95% CI)	Years	APC (95% CI)
Age-standardized incidence rates (GBD database)										
Algeria	1990–2000	10.1* (9.7, 10.5)	2001–2006	5.3* (4, 6.6)	2007–2012	1.9* (0.7, 3.2)	2013–2015	−10.4* (−15.2, −5.3)	2016–2019	−0.6 (−3.2, 2.2)
Bahrain	1990–1992	−1.6 (−5.8, 2.8)	1993–2001	−6.2* (−7, −5.3)	2002–2004	−9.2* (−16.8, −1)	2005–2019	−0.5* (−0.9, −0.1)	–	–
Djibouti	1990–1991	132.1* (120.2, 144.6)	1992–1994	63.5* (55.2, 72.4)	1995–1997	7.6* (2.1, 13.4)	1998–2002	−14* (−15.4, −12.5)	2003–2019	−5.7* (−5.9, −5.6)
Egypt	1990–1999	−3.5* (−3.8, −3.3)	2000–2009	4.8* (4.5, 5.1)	2010–2014	9 * (7.9, 10.2)	2015–2019	4.1* (3.1, 5.2)	–	–
Iran	1990–1992	0.9 (−3.3, 5.3)	1993–1998	25.7* (23.3, 28.1)	1999–2006	2.9* (1.8, 4.1)	2007–2014	12.5* (11.3, 13.8)	2015–2019	2.2 (−0.5, 5)
Iraq	1990–1993	7.3* (5.9, 8.8)	1994–1998	16* (14.4, 17.5)	1999–2008	1.8* (1.4, 2.2)	2009–2014	4.6* (3.6, 5.6)	2015–2019	0.6 (−0.8, 1.9)
Jordan	1990–1999	9.6* (9.1, 10.1)	2000–2009	1.9* (1.4, 2.4)	2010–2012	−10.2* (−15, −5.2)	2013–2019	1.9* (1, 2.9)	–	–
Kuwait	1990–1992	−8.7 (−17.8, 1.4)	1993–2000	3.3* (0.5, 6.3)	2001–2006	−8.9* (−13.1, −4.5)	2007–2019	1 (− 0.2, 2.3)	–	–
Lebanon	1990–1999	−4* (−4.6, −3.5)	2000–2002	1.1 (−6.2, 9.1)	2003–2008	−4.5* (−6.1, −2.9)	2009–2019	4.3* (3.7, 4.9)	–	–
Libya	1990–1992	6.1* (3.3, 9)	1993–1999	12.5* (11.4, 13.5)	2000–2008	−1.1* (−1.7, −0.6)	2009–2014	6.4* (5.1, 7.6)	2015–2019	2.2* (0.5, 3.9)
Morocco	1990–1999	10.8* (10.3, 11.3)	2000–2003	0 (−3, 3.2)	2004–2009	−5.5* (−6.8, −4.2)	2010–2012	−9.1* (−14.6, −3.3)	2013–2019	0 (−1.1, 1)
Oman	1990–1999	21* (18.5, 23.5)	2000–2008	−7.2* (−9.8, −4.5)	2009–2019	3.4* (1.3, 5.5)	–	–	–	–
Palestine	1990–1999	7.7* (7.4, 8.1)	2000–2002	2.3 (−1.6, 6.5)	2003–2009	−1.4* (−2.1, −0.8)	2010–2019	2.1* (1.8, 2.5)	–	–
Qatar	1990–1995	−7.1* (−7.8, −6.3)	1996–1998	1.9 (−2.7, 6.7)	1999–2002	−20.2* (−22, −18.3)	2002–2006	−8.8* (−10.9, −6.7)	2007–2019	0 (−0.3, 0.2)
Saudi Arabia	1990–1993	5.8* (4, 7.6)	1994–1998	11.3* (9.4, 13.2)	1999–2007	−3.8* (−4.4, −3.2)	2008–2013	2.2* (1, 3.4)	2014–2019	−1.5* (−2.6, −0.3)
Somalia	1990–1991	69.9* (65.4, 74.5)	1992–1994	32.6* (29.1, 36.2)	1995–1997	−1.6 (−4.2, 1.1)	1998–2003	−13.1* (−13.6, −12.6)	2004–2019	−6.8* (−6.9, −6.7)
Sudan	1990–1991	21.2* (19.1, 23.2)	1992–1994	11.5* (9.6, 13.4)	1995–1997	2.6* (0.9, 4.4)	1998–2006	−1.1* (−1.3, −0.9)	2007–2019	2.4* (2.3, 2.5)
Syria	1990–2004	−0.2 (−1.6, 1.3)	1995–2007	19.6 (−15.2, 68.7)	2008–2019	−2.9* (−5.2, −0.7)	–	–	–	–
Tunisia	1990–2001	11.9* (11.7, 12.1)	2002–2005	5.5* (3.7, 7.3)	2006–2011	0.8* (0, 1.6)	2012–2014	5* (1.5, 8.7)	2015–2019	0.4 (−0.7, 1.5)
UAE	1990–2004	9.3* (4.3, 14.6)	2005–2011	30.7* (8.1, 57.9)	2012–2019	−21.1* (−32, −8.3)	–	–	–	–
Yemen	1990–1993	−0.7 (−1.7, 0.3)	1994–1999	3.5* (2.8, 4.3)	2000–2010	0.4* (0.2, 0.7)	2011–2015	8.1* (7, 9.2)	2016–2019	0.6 (−0.9, 2.3)
Total	1990–1993	55* (49.9, 60.3)	1994–1996	18.6* (6.7, 31.7)	1997–2003	−10.6* (−12.1, −9)	2004–2019	−3.5* (−3.9, −3.1)	–	–
Incidence rates (UNAIDS database)										
Djibouti	1990–1991	78.3* (57.3, 102.1)	1992–1994	55.4* (37.1, 76.2)	1995–1997	16.1* (2.4, 31.6)	1998–2003	−23.3* (−25.4, −21.1)	2004–2019	−11.9* (−12.4, −11.5)
Egypt	1990–1993	20.3* (19.3, 21.4)	1994–2003	15.5* (15.2, 15.8)	2004–2012	14.1* (13.7, 14.4)	2013–2016	11.3* (9.8, 12.9)	2017–2019	5.5* (2.6, 8.5)
Iran	1990–1992	25.4* (17.9, 33.4)	1993–1995	59.8* (41.2, 80.8)	1996–2003	28.9* (26.8, 31.1)	2004–2012	−16* (−17.2, −14.9)	2013–2019	−6.1* (−8, −4.1)
Jordan	1990–1993	6.8* (6.1, 7.5)	1994–2001	3.9* (3.6, 4.2)	2002–2011	2.9* (2.7, 3.1)	2012–2019	2.2* (2, 2.5)	–	–
Lebanon	1990–1991	4.3* (2.3, 6.3)	1992–1997	2.3* (1.9, 2.8)	1998–2012	3* (3, 3.1)	2013–2016	−1.3* (−2.3, −0.4)	2017–2019	−4.4* (−6.2, −2.5)
Libya	1990–1998	29.7* (29.1, 30.2)	1999–2003	21* (19.2, 22.9)	2004–2006	1.9 (−2.9, 6.8)	2007–2011	−21* (−22.2, −19.8)	2012–2019	4.4* (3.8, 5.1)
Morocco	1990–1992	12.5* (10.4, 14.6)	1993–2001	4.5* (4, 4.9)	2002–2007	−1.1* (−1.9, −0.3)	2008–2012	−5.8* (−6.9, −4.7)	2013–2019	−9.1* (−9.7, −8.5)
Qatar	1990–1994	−39* (−40.9, −37.1)	1995–2000	60.1* (55.1, 65.3)	2001–2003	17* (1.5, 34.9)	2004–2012	−7.2* (−8.6, −5.8)	2013–2019	17.3* (14.5, 20.1)
Saudi Arabia	1990–1993	16.8* (13.9, 19.7)	1994–1996	3.9 (−4, 12.4)	1997–2003	9* (7.5, 10.4)	2004–2006	−7.6 (−14.6, 0)	2007–2019	8.2* (7.7, 8.7)
Somalia	1990–1994	19.9* (17.8, 22)	1995–1999	6.7* (4.1, 9.3)	2000–2004	−5.3* (−7.6, −2.9)	2005–2014	−18.3* (−18.8, −17.7)	2015–2019	−4.5* (−6.8, −2.1)
Sudan	1990–1994	46.6* (42.3, 51.1)	1995–2000	−0.5 (−3.4, 2.6)	2001–2003	−10.8 (−22.1, 2)	2004–2019	−2.5* (−3, −1.9)	–	–
Syria	1990–1999	9.3* (8.9, 9.7)	2000–2012	7.9* (7.6, 8.2)	2013–2015	1.6 (−3, 6.5)	2016–2019	−14 * (−16, −11.9)	–	–
Tunisia	1990–2001	15.1* (14.8, 15.4)	2002–2004	6.1* (1.9, 10.5)	2005–2007	−12.2* (−15.7, −8.6)	2008–2011	6.7* (4.5, 8.8)	2012–2019	3.4* (2.9, 4)
Yemen	1990–1993	4.6* (4.4, 4.7)	1994–2006	3.8* (3.8, 3.8)	2007–2010	3.4* (3.2, 3.7)	2011–2014	3* (2.8, 3.2)	2015–2019	2.3* (2.1, 2.4)

*APC* annual percentage change, *UAE* United Arab Emirates.

*P <0.05 versus 0 (output from joinpoint regression analysis).

Figure 1 presents the trends of the age-standardized incidence rates (ASIR) for HIV in MENA countries from 1990 to 2019. The ASIR of men was higher than women, except in Djibouti, Iraq, Somalia, and Sudan (Fig. 1). Table S3 presents the AAPC of HIV incidence in the region from 1990 to 2019. Among women (GBD data), seventeen countries had increases in ASIR and 4 countries showed declining trends. The highest AAPC was related to Iran (AAPC = 9.6, 95% CI 8.8, 10.5; P < 0.05) (12.3, and 8.3 in women and men, respectively). In the UNIAIDS dataset, the majority of incidence rises occurred in Egypt and Iran (P<0.05) (Table S3).

ASIR increased in almost all age groups in the region, especially those aged 44–64 years (AAPC = 3.8, 95% CI 1.4, 6.2; P < 0.05) and 25–34 years (AAPC = 3.3, 95% CI 2.6, 3.9; P < 0.05) (Table S4).

### Age-standardized mortality rate trend

**Figure 2:**
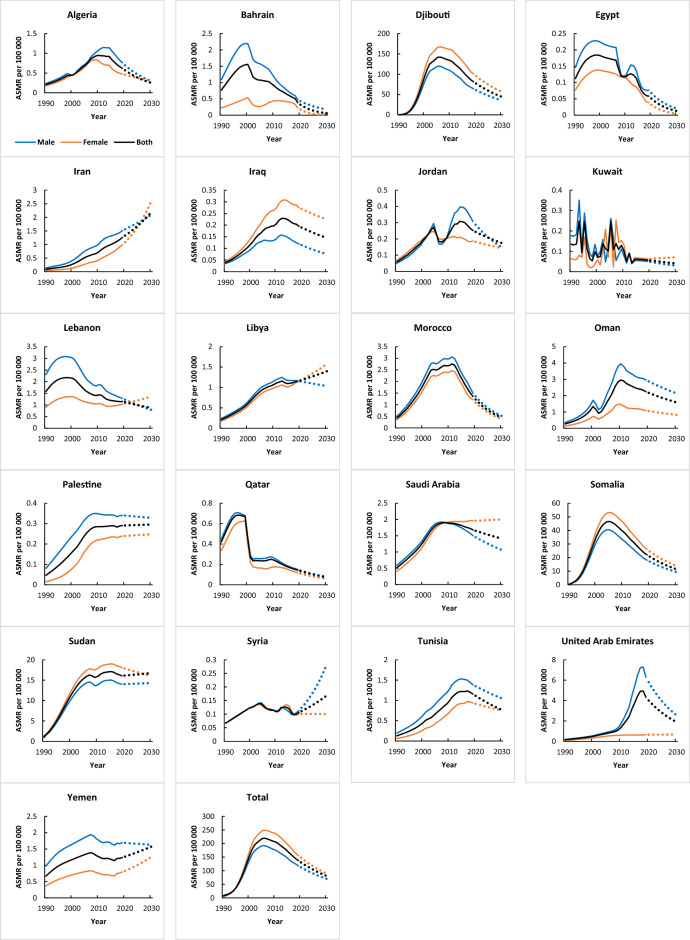
Trends in age-standardized mortality rates (1990–2019) and prediction (2020–2030) per 100,000 from HIV in MENA countries (GBD).

According to the GBD database, a total of 16 out of 21 countries reported increasing trends in the age-standardized mortality rate of HIV. Among them, Djibouti (AAPC = 24.2, 95% CI 23.0, 25.3; P < 0.001) and Somalia (AAPC = 15.1, 95% CI 14.1, 16.1 P < 0.001) showed the most marked increase in mortality rates. Qatar (AAPC = − 3.8, 95% CI − 4.3, − 3.3; P < 0.001), Kuwait (AAPC = − 3, 95% CI − 4.7, − 1.4; P < 0.001), Egypt (AAPC = − 2.7, 95% CI − 4.0, − 1.3; P < 0.001), Bahrain (AAPC = − 2.5, 95% CI − 3.6, − 1.5; P < 0.001) and Lebanon (AAPC = − 1.3, 95% CI − 1.8, − 0.8; P < 0.001) were the countries where a decrease in mortality rates was observed (Table 2 and Fig. 2). Modeling results with UNAIDS data indicate that the only country that showed decreasing mortality trends including Qatar (AAPC = − 3.8, 95% CI − 4.7, − 2.9; P < 0.001), and 13 countries showed an increasing trend in the mortality rate of HIV. The highest annual growth rate related to Iran (AAPC = 16.2, 95% CI 15.7, 16.7; P < 0.001) and the lowest annual growth rate related to Jordan (AAPC = 2.9, 95% CI 1.2, 4.7; P = 0.001) (Table 2).

**Table 2:** Joinpoint regression analysis of age-standardized mortality rates, and mortality rates from HIV in MENA countries, 1990–2019.

Country	Trend 1		Trend 2		Trend 3		Trend 4		Trend 5	
	Years	APC (95% CI)	Years	APC (95% CI)	Years	APC (95% CI)	Years	APC (95% CI)	Years	APC (95% CI)
Age-standardized mortality rates (GBD database)										
Algeria	1990–1996	10.1* (9.7, 10.4)	1997–1999	3.8* (1.3, 6.3)	2000–2007	9.5* (9.1, 9.9)	2008–2013	−0.1 (−0.7, 0.4)	2014–2019	−8* (−8.4, −7.5)
Bahrain	1990–1998	8.2* (7.3, 9.1)	1999–2002	−10.3* (−14.4, −6.1)	2003–2007	−1.2 (−4.1, 1.7)	2008–2016	−6.6* (−7.6, −5.7)	2017–2019	−15.7* (−23.2, −7.5)
Djibouti	1990–1991	243.9* (215.9, 274.3)	1992–1995	99.8* (91.5, 108.5)	1996–2000	33.7* (30.2, 37.3)	2001–2008	1.8* (0.6, 2.9)	2009–2019	−5.4* (−0.6, −4.8)
Egypt	1990–1994	9.3* (6.5, 12.2)	1995–2004	−0.3 (−1.4, 0.7)	2005–2007	−11.6* (−21.3, −0.8)	2008–2012	0.3 (−3.3, 4)	2013–2019	−13* (−14.7, −11.3)
Iran	1990–1997	10.7* (10.2, 11.1)	1998–2004	14.8* (14.1, 15.5)	2005–2008	5.1* (3.1, 7.2)	2009–2011	9.4* (5.3, 13.7)	2012–2019	5.1* (4.6, 5.7)
Iraq	1990–1995	12.5* (12.1, 13)	1996–2002	10.4* (10, 10.9)	2003–2005	5.3* (2.8, 7.8)	2006–2019	1.7* (1.5, 1.8)	–	–
Jordan	1990–1994	14.8* (13.5, 16.1)	1995–2003	9.8* (9.2, 10.4)	2004–2006	−14.5* (−18.8, −10)	2007–2012	10.8* (9.5, 10)	2013–2019	−3.3* (−4.1, −2.4)
Kuwait	–	–	–	–	–	–	–	–	–	–
Lebanon	1990–1993	6.9* (4.7, 9.2)	1994–1999	0.7 (−0.8, 2.3)	2000–2005	−5.8* (−7.3, −4.4)	2006–2019	−2.5* (−2.8, −2.1)	–	–
Libya	1990–1991	14.1* (11.8, 16.4)	1992–2003	10.2* (10.1, 10.4)	2004–2011	3.3* (3.1, 3.6)	2012–2015	−1.3* (−2.3, −0.3)	2016–2019	1.8* (0.8, 2.8)
Morocco	1990–1993	19.2* (18.2, 20.3)	1994–1998	13.8* (12.8, 14.9)	1999–2002	10.5* (9, 12.1)	2003–2011	1.2* (0.9, 1.5)	2012–2019	−9.6* (−9.9, −9.2)
Oman	1990–1999	16.7* (16.3, 17)	2000–2002	−6.3* (−10.1, −2.3)	2003–2008	20* (18.9, 21.1)	2009–2019	−3.1* (−3.4, −2.8)	–	–
Palestine	1990–1992	17.5* (16.3, 18.6)	1993–1996	12.5* (11.4,13.6)	1997–2004	9.3* (9, 9.6)	2005–2007	4.4* (2.3, 6.5)	2008–2019	0.2* (0, 0.3)
Qatar	1990–1994	9.9* (9, 10.9)	1995–1998	−1.8 (−3.7, 0.1)	1999–2001	−30.1* (−32.7, −27.4)	2002–2008	1.8* (1.2, 2.5)	2009–2019	−5.6* (−5.9, −5.3)
Saudi Arabia	1990–1992	11.6* (11.1, 12.1)	1993–2002	8.8* (8.7, 8.9)	2003–2005	4.1* (3.1, 5.1)	2006–2011	0 (− 0.2, 0.2)	2012–2019	−1.6* (−1.7, −1.5)
Somalia	1990–1991	153.6* (135.8, 172.6)	1992–1995	55.7* (50.2, 61.5)	1996–1999	26.8* (22.3, 31.4)	2000–2005	4.5* (2.8, 6.2)	2006–2019	−5.8* (−6.1, −5.4)
Sudan	1990–1991	49.9* (39.8, 60.8)	1992–1995	27.1* (22.7, 31.6)	1996–1999	14.5* (10.5, 18.6)	2000–2004	6.8* (4.4, 9.2)	2005–2019	0.4* (0, 0.7)
Syria	1990–2001	6* (5.5, 6.5)	2002–2009	−3.1* (−4.1, −2.1)	2010–2013	4.3* (0.2, 8.5)	2014–2016	−9.1* (−16.1, −1.6)	2017–2019	4.3* (−3.7, 12.9)
Tunisia	1990–1992	15.3* (13.7, 16.9)	1993–2000	12.3* (11.9, 12.8)	2001–2011	7.8* (7.6, 8.1)	2012–2016	1.5* (0.6, 2.4)	2017–2019	−3.7* (−6.3, −1)
UAE	1990–2000	11.5* (11, 12)	2001–2008	8.6* (7.6, 9.6)	2009–2013	24.1* (21.5, 26.7)	2014–2016	17.3* (9.9, 25.3)	2017–2019	−7.3* (−13.2, −0.9)
Yemen	1990–1994	8.3* (7.6, 9)	1995–2006	2.7* (2.5, 2.9)	2007–2010	−3.5* (−4.9, −2.1)	2011–2015	−0.9* (−1.8, 0)	2016–2019	2.2* (0.7, 3.7)
Total	1990–1997	39.5* (38.7, 40.3)	1998–2000	20.1* (14.1, 26.5)	2001–2004	5.1* (2.4, 7.8)	2005–2010	−1.4* (−2.5, −0.2)	2011–2019	−4.7* (−5.3, −4.2)
Mortality rates (UNAIDS database)										
Djibouti	1990–1994	78.4* (74.3, 82.5)	1995–1999	32.6* (28.3, 36.9)	2000–2005	6.6* (4.2, 9.1)	2006–2012	−5* (−6.7, −3.4)	2013–2019	−14* (−15.5, −12.5)
Egypt	1990–1994	35.5* (32.9, 38.1)	1995–2003	21.1* (20, 22.2)	2004–2006	−1.2 (−9.4, 7.6)	2007–2013	12.4* (10.8, 14.1)	2014–2019	1.8 (−0.2, 3.7)
Iran	1990–1994	19.2* (18.1, 20.3)	1995–2004	33.4* (32.9, 33.9)	2005–2008	18.9* (16.6, 21.3)	2009–2013	5.1* (3.8, 6.4)	2014–2019	−6.8* (−7.6, −5.9)
Jordan	1990–1998	7.6* (6.1, 9.2)	1999–2001	−16.2* (_28.3, −2.1)	2002–2014	6.2* (5.2, 7.2)	2015–2019	−1.8 (−6.6, 3.1)	–	–
Lebanon	1990–1992	25.2* (20.7, 29.9)	1993–1996	14.3* (10.2, 18.5)	1997–1999	6 (−1.5, 14.1)	2000–2002	−10.5* (−16.8, −3.7)	2003–2019	0.8* (0.5, 1)
Libya	1990–1998	31.6* (30.5, 32.8)	1999–2001	−0.4 (−9.4, 9.6)	2002–2006	21.3* (17.7, 25)	2007–2013	2.8* (1.1, 4.4)	2014–2019	−3.8* (−5.8, −1.8)
Morocco	1990–1994	18.8* (16.2, 21.4)	1995–2000	12.9* (10.5, 15.4)	2001–2010	1* (0.1, 1.9)	2011–2019	−8.5* (−9.4, −7.5)	–	–
Qatar	1990–1993	11.3* (8.7, 13.9)	1994–2003	−5.9* (−6.5, −5.3)	2004–2013	9.6* (8.9, 10.3)	2014–2016	−39.6* (−43.9, −34.9)	2017–2019	−15.8* (−21.9, −9.3)
Saudi Arabia	1990–1993	28.1* (23.8, 32.6)	1994–1996	−13.1* (−22, −3.1)	1997–2004	11.1* (9.5, 12.7)	2005–2013	−1 (−2.1, 0.2)	2014–2019	10.5* (7.8, 13.2)
Somalia	1990–1994	29.2* (27.7, 30.6)	1995–1999	18.3* (16.5, 20.2)	2000–2005	8.1* (6.9, 9.3)	2006–2012	−5.1* (−5.9, −4.3)	2013–2019	−12.2* (−12.9, −11.4)
Sudan	1990–1995	45.9* (42.4, 49.4)	1996–1999	27.1* (18.3, 36.4)	2000–2004	11.8* (6.9, 17)	2005–2015	−1.6* (−2.7, −0.6)	2016–2019	−9.2* (−15.4, −2.5)
Syria	1990–2000	8.8* (7.7, 9.8)	2001–2012	5.1* (4.1, 6.1)	2013–2016	12.2* (4.4, 20.5)	2017–2019	− 7.7 (− 20, 6.6)	–	–
Tunisia	1990–1999	17.6* (16.9, 18.3)	2000–2002	3.1 (−4.4, 11.2)	2003–2005	15.4* (7, 24.5)	2006–2014	6.7* (5.8, 7.6)	2015–2019	−0.5 (−2.8, 1.9)
Yemen	1990–1994	9.5* (7.9, 11)	1995–2006	4.1* (3.6, 4.5)	2007–2009	−9.4* (−15, −3.4)	2010–2019	2.5* (1.9, 3.1)	–	–

*APC* annual percentage change, *UAE* United Arab Emirates.

*P <0.05 versus 0 (output from joinpoint regression analysis).

A total of 17 and 18 out of 21 reported increasing mortality trends in men and women, respectively. Among them, Djibouti (AAPC_men_ = 23.3, 95% CI 21.5, 25, AAPC _women_ = 24.6, 95%CI 23.4, 25.9; P < 0.05) showed the most marked increase in mortality rates. In UNIAIDS data, Iran had the greatest mortality increase (AAPC = 16.2, 95% CI 16, 16.4; P < 0.05) (Table S5). Table S6 presents the AAPC of mortality rates from HIV in MENA countries by age groups, 1990–2019.

### Disability-adjusted life years (DALY) trend

**Figure 3:**
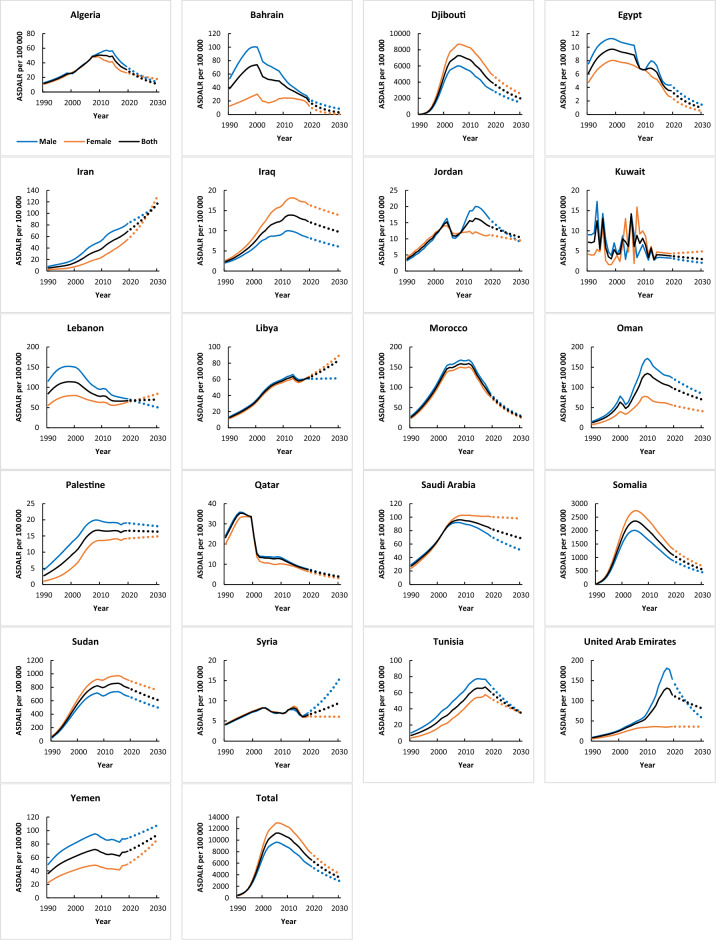
Trends in disability-adjusted life years (1990–2019) and prediction (2020–2030) per 100,000 from HIV in MENA countries (GBD).

The DALY trends of each country were shown in Table 3 and Fig. 3, and the corresponding findings from the joinpoint regression analysis were presented in Tables S7 and S8. According to GBD datasets, 16 countries had an increasing pattern in DALY and five countries showed declining trends in both genders between 1990 and 2019 (Table S7).

**Table 3:** Joinpoint regression analysis of disability-adjusted life years from HIV in MENA countries, 1990–2019.

Country	Trend 1		Trend 2		Trend 3		Trend 4		Trend 5	
	Years	APC (95% CI)	Years	APC (95% CI)	Years	APC (95% CI)	Years	APC (95% CI)	Years	APC (95% CI)
Age-standardized rates disability-adjusted life years (GBD database)										
Algeria	1990–1996	9.8* (9.2, 11)	1997–1999	5.8* (4.8, 8)	2000–2007	8.6 (−0.1, 10)	2008–2013	−1.3* (−2.8, −0.4)	2014–2019	−9.1* (−10.1, −8.1)
Bahrain	1990–1997	8.6* (7.4, 9.7)	1998–2003	−6.6* (−9.3, −4.5)	2004–2007	−1.4 (−4.1, 1.4)	2008–2016	−6.4* (−7.4, −5.3)	2017–2019	−15.2* (−18.7, −10)
Djibouti	1990–1991	211.3* (190.9, 235.5)	1992–1995	93.1* (87.4, 99.1)	1996–2000	31.3* (28.2, 34.3)	2001–2008	1.3 (−0.2, 3.2)	2009–2019	−6.1* (−7.3, −5)
Egypt	1990–1994	8.5* (6.4, 11.4)	1995–2004	−0.4 (−1.1, 0.6)	2005–2007	−9.4* (−11.4, −5.6)	2008–2012	−0.4 (−2.4, 4)	2013–2019	−11.3* (−12.8, −9.8)
Iran	1990–1996	9.2* (8.7, 9.6)	1997–2004	14.4* (14, 14.8)	2005–2008	5.8* (4.7, 6.5)	2009–2011	9.2* (7.8, 10)	2012–2019	5* (4.5, 5.4)
Iraq	1990–2001	11.7* (11.5, 11.9)	2002–2005	6.6* (5.9, 7.8)	2006–2012	3* (2.6, 3.4)	20132019	−2.1* (−2.5, −1.7)	–	–
Jordan	1990–1996	13* (11.9, 14.6)	1997–2003	8* (6.7, 9)	2004–2006	−12.1* (−13.8, −10.1)	2007–2012	8* (6.6, 9.5)	2013–2019	−2.6* (−3.9, −1.2)
Kuwait	–	–	–	–	–	–	–	–	–	–
Lebanon	1990–1993	6.6* (5.1, 9)	1994–1999	0.7 (−0.6, 1.8)	2000–2005	−4.8* (−6.8, −4.1)	2006–2014	−2.8* (−3.4, −2.1)	2015–2019	0.4 (−0.9, 3)
Libya	1990–2002	10.2* (10, 10.4)	2003–2005	5.7* (4.3, 8.3)	2006–2012	2.2* (1.8, 2.6)	2013–2015	−3* (−3.8, −1.6)	2016–2019	2.8* (1.7, 5)
Morocco	1990–1993	18.7* (17.6, 20.1)	1994–1998	13.6* (12.7, 14.8)	1999–2002	10.7* (7.6, 11.4)	2003–2011	0.8* (0.5, 1)	2012–2019	−9.3* (−9.6, −9)
Oman	1990–1999	16.8* (14.2, 18.8)	2000–2002	−5.2 (−7.9, 18.9)	2003–2005	22.1 (−5, 26.5)	2006–2008	13.6 (−5.9, 18.2)	2009–2019	−3.1* (−6.6, −0.5)
Palestine	1990–1992	16.5* (15.3, 18.1)	1993–1996	12.6* (11.3, 13.6)	1997–2004	10* (9.5, 10.3)	2005–2007	4* (2.7, 5.4)	2008–2019	−0.2 (−0.4, 0)
Qatar	1990–1994	8.9* (6.6, 12.7)	1995–1998	−2.9 (−5.5, 0)	1999–2001	−26.7* (−28.9, −21.8)	2002–2008	0.2 (−1.2, 2.7)	2009–2019	−5.6* (−6.8, −4.8)
Saudi Arabia	1990–1992	10.6* (10, 11.3)	1993–2002	8.8* (8.7, 8.9)	2003–2005	3.6* (3.3, 3.9)	2006–2011	−0.4* (−0.6, −0.1)	2012–2019	−1.7* (−1.9, −1.5)
Somalia	1990–1991	132.2* (112.1, 149.2)	1992–1995	53.2* (46.2, 61.7)	1996–1999	24.6* (15.8, 32.8)	2000–2005	3.7 (−0.1, 7.8)	2006–2019	−5.9* (−6.8, −5.1)
Sudan	1990–1992	41.7* (39, 44.5)	1993–1997	21* (19.5, 22.4)	1998–2003	8.2* (7.1, 9.5)	2004–2014	0.8* (0.4, 1.5)	2015–2019	−2.3* (−5.1, −0.7)
Syria	1990–2001	5.6* (5.1, 6.1)	2002–2009	−2.7* (−3.9, −1.7)	2010–2013	5.9* (3.9, 9.2)	2014–2016	−10.7* (−12.7, −8)	2017–2019	3.5 (−0.8, 7.3)
Tunisia	1990–1992	14.9* (13.6, 16.7)	1993–2000	12.5* (11.9, 12.8)	2001–2011	7.7* (7.5, 7.8)	2012–2016	1.4* (1, 1.8)	2017–2019	−4.9* (−6.2, −2.9)
UAE	1990–1992	13.7* (11.5, 16.9)	1993–2002	10.9* (7.6, 11.3)	2003–2008	7.1* (5.3, 9.1)	2009–2015	13.5* (12.8, 14.5)	2016–2019	−3.1* (−4.6, −1.7)
Yemen	1990–1994	7.7* (6.7, 8.9)	1995–2005	2.8* (2.4, 3.1)	2006–2014	−1.6* (−2.2, −1.2)	2015–2019	2.8* (1.5, 5)	–	–
Total	1990–1996	40* (39.1, 41.2)	1997–1999	25.4* (22.3, 29.2)	2000–2003	7.6* (5.6, 9.4)	2004–2010	−1.4* (−2.2, −0.4)	2011–2019	−5.5* (−6.3, −4.9)

*APC* annual percentage change, *UAE* United Arab Emirates.

*P < 0.05 versus 0 (output from joinpoint regression analysis).

### Projection of incidence, mortality rates, and DALY

Tables 4, 5 and 6 summarize the projected age-standardized incidence, mortality rates, and DALY of HIV by 2025 and 2030 per 100,000 in the countries in the region. The estimated increase in the incidence by 2030 was most marked in Egypt (339%), based on UNAIDS estimates. However, the estimated higher age-standardized incidence rate by 2030 was found in Iran (174%), according to estimates from GBD. Countries with substantial growth rates of age-standardized incidence rates and incidence rates between 2019 and 2030 include Lebanon (59%) and Qatar (478%), respectively (Table 4 and Fig. 1).

**Table 4:** Age-standardized incidence rates and prediction of age-standardized incidence rates from HIV in 2025 and 2030 per 100000 in MENA countries.

Country	Age-standardized incidence rates (GBD database)											Incidence rates (UNAIDS database)										
	Available				Prediction		Growth rate (%)					Available				Prediction		Growth rate (%)				
	1990	2000	2010	2019	2025	2030	1990–2019	1990–2025	1990–2030	2019–2025	2019–2030	1990	2000	2010	2019	2025	2030	1990–2019	1990–2025	1990–2030	2019–2025	2019–2030
Algeria	0.53	1.37	2.19	1.61	1.56	1.52	207	197	189	−3	−6	–	–	–	–	–	–	–	–	–	–	–
Bahrain	2.31	1.38	0.98	0.88	0.85	0.83	−62	−63	−64	−3	−5	–	–	–	–	–	–	–	–	–	–	–
Djibouti	12.65	280.46	119.55	69.86	48.99	36.44	452	287	188	−30	−48	23.8	274.42	44.36	13.79	6.43	3.40	−42	−73	−86	−53	−75
Egypt	0.27	0.19	0.29	0.53	0.68	0.83	99	154	211	28	56	0.06	0.31	1.2	3.04	4.18	5.47	4960	6873	9009	38	80
Iran	0.29	1.17	1.96	3.91	4.46	4.97	1264	1455	1635	14	27	0.33	7.61	6.73	2.9	1.99	1.45	788	510	346	−31	−50
Iraq	0.13	0.37	0.45	0.57	0.59	0.61	337	352	365	3	6	–	–	–	–	–	–	–	–	–	–	–
Jordan	0.2	0.48	0.57	0.46	0.52	0.57	136	165	192	12	24	0.29	0.47	0.65	0.8	0.92	1.02	175	214	250	14	28
Kuwait	0.45	0.43	0.26	0.28	0.30	0.32	−37	−34	−30	6	12	–	–	–	–	–	–	–	–	–	–	–
Lebanon	2.98	2.05	1.66	2.38	3.06	3.78	−20	3	27	29	59	1.66	2.21	2.98	2.83	2.16	1.73	70	30	4	−24	−39
Libya	0.62	1.67	1.63	2.4	2.74	3.05	286	339	389	14	27	0.4	5.1	5.55	4.97	6.44	7.99	1145	1514	1903	30	61
Morocco	1.66	4.8	3.48	2.58	2.57	2.56	55	54	54	0	0	2.69	5.26	4.74	2.17	1.23	0.76	−19	−54	−72	−44	−65
Oman	1.09	6.29	3.93	4.42	5.39	6.37	306	395	484	22	44	–	–	–	–	–	–	–	–	–	–	–
Palestine	0.18	0.36	0.36	0.42	0.48	0.53	141	173	204	14	26	–	–	–	–	–	–	–	–	–	–	–
Qatar	1.05	0.59	0.2	0.2	0.20	0.20	−81	−81	−81	0	0	1.91	1.72	2.38	5.61	14.60	32.41	194	666	1601	160	478
Saudi Arabia	1.55	3.2	2.45	2.46	2.25	2.09	58	45	35	−8	−15	0.63	1.69	2.35	4.62	7.43	11.04	637	1086	1661	61	139
Somalia	16.07	78.25	29.42	15.95	10.45	7.35	−1	−35	−54	−34	−54	6.23	21.01	5.97	1.88	1.43	1.13	−70	−77	−82	−24	−40
Sudan	13.84	30.01	29.55	36.46	42.02	47.31	163	204	242	15	30	2.83	17.7	10.75	9.1	7.82	6.90	222	176	144	−14	−24
Syria	0.13	0.17	0.24	0.21	0.18	0.15	58	32	14	−16	−28	0.07	0.17	0.37	0.32	0.13	0.06	345	81	−15	−59	−81
Tunisia	0.45	1.38	2.2	2.6	2.66	2.72	476	490	502	2	4	0.45	1.89	2.32	3.28	4.01	4.74	627	789	951	22	45
UAE	0.51	1.45	6.47	3.76	0.91	0.28	634	77	−46	−76	− 93	–	–	–	–	–	–	–	–	–	–	–
Yemen	1.54	1.84	1.91	2.94	3.06	3.16	91	99	105	4	7	1.28	1.92	2.75	3.5	4.01	4.48	173	213	250	14	28
Total	58.50	417.91	209.74	154.90	124.96	104.48	165	114	79	−19	−33	–	–	–	–	–	–	–	–	–	–	–

*UAE* United Arab Emirates.

**Table 5:** Age-standardized mortality rates and prediction of age-standardized mortality rates from HIV in 2025 and 2030 per 100000 in MENA countries.

Country	Age-standardized mortality rates (GBD database)											Mortality rates (UNAIDS database)										
	Available				Prediction		Growth rate (%)					Available				Prediction		Growth rate (%)				
	1990	2000	2010	2019	2025	2030	1990–2019	1990–2025	1990–2030	2019–2025	2019–2030	1990	2000	2010	2019	2025	2030	1990–2019	1990–2025	1990–2030	2019–2025	2019–2030
Algeria	0.21	0.44	0.95	0.62	0.38	0.25	201	83	21	−39	−60	–	–	–	–	–	–	–	–	–	–	–
Bahrain	0.77	1.56	0.9	0.38	0.14	0.06	−50	−82	−92	−64	−85	–	–	–	–	–	–	–	–	–	–	–
Djibouti	0.17	94.32	133.57	81.48	58.40	44.24	48364	34633	26214	−28	−46	1.08	77.9	102.79	35.9	14.56	6.86	3229	1250	536	−59	−81
Egypt	0.11	0.18	0.12	0.06	0.03	0.01	−48	−78	−89	−57	−78	0	0.06	0.17	0.27	0.30	0.33	6094	6778	7405	11	21
Iran	0.09	0.27	0.72	1.26	1.71	2.19	1302	1795	2335	35	74	0.06	0.59	5.25	4.43	2.91	2.05	7386	4821	3369	−34	−54
Iraq	0.04	0.12	0.2	0.23	0.17	0.14	400	325	271	–15	–26	–	–	–	–	–	–	–	–	–	–	–
Jordan	0.06	0.19	0.23	0.25	0.21	0.17	332	254	200	–18	–31	0.09	0.17	0.17	0.22	0.20	0.18	142	117	98	–10	–18
Kuwait	0.13	0.07	0.1	0.06	0.05	0.04	–58	–65	–70	–17	–29	–	–	–	–	–	–	–	–	–	–	–
Lebanon	1.58	2.15	1.43	1.15	0.99	0.87	–27	–38	–45	–14	–24	0.27	1.11	0.84	0.86	0.90	0.93	213	227	240	5	9
Libya	0.21	0.6	1.08	1.15	1.28	1.40	446	507	563	11	21	0.04	0.51	1.3	1.28	1.01	0.83	3184	2499	2039	–21	–35
Morocco	0.43	1.85	2.69	1.36	0.74	0.45	218	73	5	–45	–67	0.48	2.08	2.6	1.26	0.74	0.47	161	54	–1	–41	–62
Oman	0.26	1.33	2.96	2.24	1.85	1.58	748	602	500	–17	–29	–	–	–	–	–	–	–	–	–	–	–
Palestine	0.05	0.16	0.29	0.29	0.29	0.30	522	528	533	1	2	–	–	–	–	–	–	–	–	–	–	–
Qatar	0.42	0.45	0.24	0.14	0.10	0.08	–66	–76	–82	–29	–47	1.07	1.16	1.61	0.37	0.13	0.06	–65	–88	–95	–64	–85
Saudi Arabia	0.52	1.3	1.89	1.68	1.53	1.41	224	195	172	–9	–16	0.07	0.18	0.26	0.42	0.77	1.27	524	1033	1763	82	199
Somalia	0.35	35.01	39.34	22.01	15.39	11.42	6270	4354	3205	–30	–48	0.72	5.9	8.18	3.12	1.43	0.75	335	99	4	–54	–76
Sudan	1.12	11.23	15.93	16.08	16.44	16.75	1330	1362	1389	2	4	0.19	4.68	7.04	5.48	3.07	1.89	2716	1475	871	–44	–66
Syria	0.07	0.12	0.11	0.11	0.14	0.17	59	105	152	29	58	0.02	0.05	0.09	0.13	0.08	0.06	523	287	160	–38	–58
Tunisia	0.13	0.45	0.99	1.16	0.92	0.77	796	615	493	–20	–34	0.08	0.42	0.9	1.26	1.22	1.19	1404	1361	1327	–3	–5
UAE	0.16	0.49	1.35	4.32	2.75	1.89	2597	1616	1077	–36	–56	–	–	–	–	–	–	–	–	–	–	–
Yemen	0.67	1.18	1.26	1.24	1.41	1.57	85	110	134	14	27	0.43	0.84	0.87	1.03	1.20	1.36	138	177	213	16	31
Total	7.54	153.47	206.35	137.22	102.51	80.40	1719	1259	966	–25	–41	–	–	–	–	–	–	–	–	–	–	–

*UAE* United Arab Emirates.

**Table 6:** Disability-adjusted life years and prediction of disability-adjusted life years from HIV in 2025 and 2030 per 100000 in MENA countries.

Country	Disability-adjusted life years (GBD database)										
	Available				Prediction		Growth rate (%)				
	1990	2000	2010	2019	2025	2030	1990–2019	1990–2025	1990–2030	2019–2025	2019–2030
Algeria	11.67	26.08	50.43	30.64	17.30	10.74	162	48	−8	−44	−65
Bahrain	38.39	73.76	43.02	18.73	6.96	3.05	−51	−82	−92	−63	−84
Djibouti	13.07	5034.22	6749.16	3863.82	2656.56	1944.19	29472	20232	14780	−31	−50
Egypt	6.16	9.51	6.63	3.52	1.72	0.95	−43	−72	−85	−51	−73
Iran	5.39	15.15	40.53	69.04	92.59	118.24	1182	1619	2096	34	71
Iraq	2.32	7.06	12.67	12.28	10.79	9.70	430	366	319	−12	−21
Jordan	3.66	11.52	13.26	13.88	11.85	10.39	279	223	184	−15	−25
Kuwait	7.15	4.16	6.52	3.76	3.30	2.97	−47	−54	−59	−12	−21
Lebanon	84.37	112.86	79.02	67.22	68.72	69.99	−20	−19	−17	2	4
Libya	12.66	34.04	59.44	62.09	73.27	84.12	391	479	565	18	35
Morocco	26.16	109.78	157.02	79.73	44.47	27.34	205	70	5	−44	−66
Oman	12.86	63.61	134.62	98.93	81.66	69.59	669	535	441	−17	−30
Palestine	2.73	9.37	16.68	16.67	16.49	16.35	511	505	499	−1	−2
Qatar	23.24	23.51	12.32	7.30	5.15	3.85	−69	−78	−83	−29	−47
Saudi Arabia	27.43	67.03	94.63	82.84	74.78	68.68	202	173	150	−10	−17
Somalia	25.31	1839.48	1963.58	1087.26	754.22	556.08	4196	2880	2097	−31	−49
Sudan	64.08	580.63	806.73	786.51	684.77	610.11	1127	969	852	−13	−22
Syria	4.13	7.42	6.84	6.52	8.00	9.49	58	94	130	23	46
Tunisia	7.11	24.71	53.83	60.73	45.02	35.08	754	533	394	−26	−42
UAE	8.57	26.03	60.81	114.14	94.42	80.62	1232	1002	841	−17	−29
Yemen	36.13	61.71	66.80	69.27	81.94	94.26	92	127	161	18	36
Total	422.57	8141.65	10434.52	6554.88	4674.17	3526.31	1451	1006	734	−29	−46

*UAE* United Arab Emirates.

Most countries in the region showed increasing mortality rates, except Qatar (35%), Kuwait (28%), Egypt (26%), Bahrain (24%), and Lebanon (13%) which showed decreased rates. Djibouti (985%) and Egypt (339%) showed a prominent rise in mortality rates by 2030, according to estimates from GBD and UNAIDS, respectively. Table 5 shows the prediction and growth rate of age-standardized mortality rates and mortality rates between 1990 and 2030 (Table 5 and Fig. 2).

**Figure 4:**
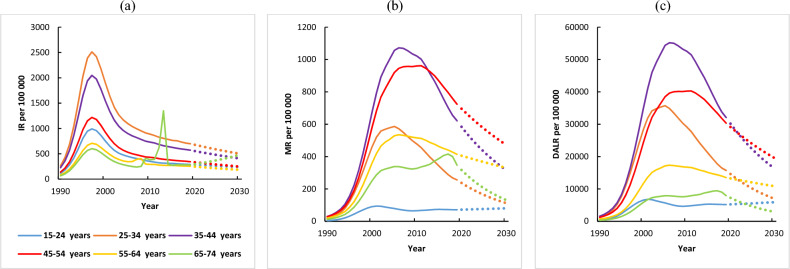
Total trends in (**a**) incidence, (**b**) mortality, and (**c**) disability-adjusted life years (1990–2019) and prediction (2020–2030) per 100,000 from HIV in MENA countries (GBD).

The estimated increase in DALY by 2030 was most marked in Djibouti, Sudan, Somalia, and Iran. Moreover, Iran (71%), Syria (46), Yemen (36), and Libya (35%) have the highest growth rate of DALY between 2019 and 2030 (Table 6 and Fig. 3). Additionally, the trends in incidence, mortality, and DALY (1990–2019) and their prediction (2019–2030) in the region are presented in Fig. 4a–c.

### APC model analysis results of HIV incidence, mortality rates, and DALY

Table 7 shows the estimated impact of age, period, and birth cohort of the whole population on the age-standardized incidence rate, mortality rates, and DALY of HIV in the region. The IE algorithm was used for quantitative analysis among different age groups and periods.

**Table 7:** The age-period-cohort model analysis results of HIV incidence, mortality, and disability-adjusted life years in MENA countries (GBD).

Index	Age				Period				Cohort			
	Range	Coefficient	SE	P value	Range	Coefficient	SE	P value	Range	Coefficient	SE	P value
Incidence	15–24 years	**–**	**–**	**–**	1990–94	**–**	**–**	**–**	1960–64	0.053	0.048	<0.001
	25–34 years	**–**	**–**	**–**	1995–99	**–**	**–**	**–**	1965–69	−0.028	0.030	<0.001
	35–44 years	−1.113	0.015	<0.001	2000–04	−1.460	0.015	<0.001	1970–74	−0.019	0.023	<0.001
	45–54 years	−0.266	0.015	<0.001	2005–09	0.093	0.016	<0.001	1975–79	−0.199	0.017	<0.001
	55–54 years	0.110	0.019	<0.001	2010–14	0.315	0.018	<0.001	1980–84	0.064	0.016	<0.001
	65–74 years	0.496	0.023	<0.001	2015–19	−0.140	0.020	<0.001	1985–89	−0.027	0.018	<0.001
	–	**–**	**–**	**–**	–	**–**	**–**	**–**	1990–94	−0.009	0.021	<0.001
	–	**–**	**–**	**–**	–	**–**	**–**	**–**	1995–99	0.018	0.026	<0.001
	–	**–**	**–**	**–**	–	**–**	**–**	**–**	2000–04	0.052	0.039	<0.001
Mortality	15–24 years	**–**	**–**	**–**	1990–94	**–**	**–**	**–**	1960–64	−0.039	0.117	<0.001
	25–34 years	**–**	**–**	**–**	1995–99	**–**	**–**	**–**	1965–69	0.020	0.054	<0.001
	35–44 years	−1.102	0.033	<0.001	2000–04	−0.772	0.041	<0.001	1970–74	0.050	0.034	<0.001
	45–54 years	−0.627	0.019	<0.001	2005–09	−0.712	0.022	<0.001	1975–79	0.031	0.025	<0.001
	55–54 years	−0.460	0.019	<0.001	2010–14	−0.173	0.019	<0.001	1980–84	−0.076	0.020	<0.001
	65–74 years	0.253	0.024	<0.001	2015–19	−0.040	0.019	<0.001	1985–89	−0.088	0.020	<0.001
	–	**–**	**–**	**–**	–	**–**	**–**	**–**	1990–94	−0.155	0.024	<0.001
	–	**–**	**–**	**–**	–	**–**	**–**	**–**	1995–99	−0.057	0.036	<0.001
	–	**–**	**–**	**–**	–	**–**	**–**	**–**	2000–04	0.075	0.039	<0.001
Disability-adjusted life years	15–24 years	**–**	**–**	**–**	1990–94	**–**	**–**	**–**	1960–64	−0.023	0.023	<0.001
	25–34 years	**–**	**–**	**–**	1995–99	**–**	**–**	**–**	1965–69	0.022	0.010	<0.001
	35–44 years	−1.108	0.004	<0.001	2000–04	−0.784	0.005	<0.001	1970–74	0.051	0.006	<0.001
	45–54 years	−0.662	0.002	<0.001	2005–09	−0.677	0.003	<0.001	1975–79	0.028	0.004	<0.001
	55–54 years	−0.505	0.003	<0.001	2010–14	−0.159	0.002	<0.001	1980–84	−0.071	0.003	<0.001
	65–74 years	0.179	0.004	<0.001	2015–19	−0.052	0.002	<0.001	1985–89	−0.114	0.002	<0.001
	–	**–**	**–**	**–**	**–**	**–**	**–**	**–**	1990–94	−0.146	0.003	<0.001
	**–**	**–**	**–**	**–**	**–**	**–**	**–**	**–**	1995–99	−0.040	0.004	<0.001
	**–**	**–**	**–**	**–**	**–**	**–**	**–**	**–**	2000–04	0.495	0.009	<0.001

The age effect of HIV incidence, mortality rates, and DALY showed a net decrease of 0.83, 0.29, and 0.01 from the age of 35–74 years (the lowest value 35–44 years age group as a reference). According to period effects, there is a significant upward trend in the risk of incidence, mortality rates, and DALY from HIV among the total population. According to the analysis of cohort effects, the incidence, mortality rates, and DALY from HIV have no clear or consistent trend. The 1972–1975 period had the lowest cohort effect on incidence risk from HIV. Also, the 1990–1994 period had the lowest cohort effect on mortality and DALY risk from HIV in the region (Table 7).

## Discussion

Our study showed that the incidence and mortality of HIV have increased in most countries in the MENA region from 1990 to 2019. Iran and Egypt showed the highest annual growth rates in incidence, while Qatar and Somalia experienced substantial reductions in incidence based on the modeling results using GBD and UNAIDS estimates, respectively. Djibouti and Iran had the highest mortality rates according to the modeling results using GBD and UNAIDS estimates, respectively, whereas Qatar had the lowest mortality rate according to both datasets. Moreover, incidence trends from 21 countries in a period of 30 years reported that a total of 14 and 11 countries experienced increases in the annual growth rate according to the GBD and UNAIDS estimates, respectively. Up to five out of 21 countries showed a reduction in mortality trends according to GBD information, with Qatar being the only country to demonstrate a significant decrease in mortality rate according to both estimates. Regarding the incidence rate, Iran, Egypt, Tunisia, Libya, and Djibouti exhibited the highest percentage of change from 1990 to 2019, while Lebanon, Morocco, Syria, and Somalia reported the lowest percentage of change in HIV incidence. A higher percentage of change in the HIV mortality rates is found in Sudan, UAE, Somalia, and Djibouti, while Syria, Lebanon, and Bahrain reported the lowest percentage of change in the mortality rate of HIV. We also found an increasing pattern of DALY in most countries in the region.

Our findings showed that most countries in the region encountered an increase in the incidence of HIV from 1990 to 2019 which is consistent with other studies^9,18–21^, while the global incidence of the disease was decreasing^19,20,22^. However, the incidence rate of HIV in MENA countries was lower than the global incidence rate^22^. A study, which followed the trend of HIV in the MENA region from 1999 to 2017, showed that the average global incidence rate of HIV declined dramatically from 36.52 to 25.05 per 100,000 population. The incidence rate of HIV in the countries of MENA was lower than the mean global incidence rates. In all other countries of the region except Sudan, the incidence, prevalence, mortality, and DALY rates were reported to be lower than the global average^20^. However, it is important to note that harm reduction and HIV prevention programs in most MENA countries have faced significant challenges due to security crises, as well as political, social, and financial obstacles^23^. For example, several countries in the region, such as Afghanistan, Iraq, Libya, Syria, and Yemen, have experienced an influx of refugees as a result of ongoing conflicts. The conflict in Syria, in particular, has placed a significant burden on neighboring countries like Lebanon, Jordan, and, to a lesser extent, Egypt. It is crucial to acknowledge that substance use, including injecting drug use, among refugee and immigrant populations is often neglected within the realm of public health, despite being recognized as an important risk environment for substance-related harm, including HIV transmission^23,24^. According to the World Health Organization (WHO), a total of 83 countries worldwide have repressive laws that create barriers to the HIV response. Additionally, 33 countries and territories still have the death penalty for drug offenses, with 16 of these countries located in the MENA region^25^. Furthermore, in the MENA region, the availability and validity of data on FSWs and MSMs are hindered by various barriers, including stigma and other factors^20,26,27^. Thus, making direct comparisons between countries in the region and those in Europe and North America becomes challenging due to these data limitations.

In this study, based on the GBD data, the incidence trend was increasing in 14 out of 21 countries, stable in five, and decreasing in only two countries. Also, according to UNAIDS data, there was an increase in 11 out of 14 countries and a decrease in 2 countries. These findings suggest that a significant proportion of countries in the region may lack effective programs to reduce HIV incidence or face challenges in implementing existing programs successfully. On the other hand, the increase in incidence can be attributed, at least in part, to improved diagnosis in these countries. In any case, these countries are likely experiencing a combination of different situations, and all aspects of each country need to be examined to obtain an accurate result. While the incidence trend decreased in Qatar and Bahrain during the whole period, it increased in Iran, Egypt, Iraq, Libya, Palestine, Tunisia, and Yemen. In the rest of the countries, the trend was unsteady. The significant decreasing trend we observed in Qatar was consistent with findings from a cohort study during a 17-year period, which demonstrated the lower incidence rate of HIV in comparison to the world average^28^. Shakiba, et al., reported Qatar as the country with the lowest HIV burden in 2017 in the region^20^. The declining trend of the HIV incidence rate in Qatar coincided with the implementation of diagnostic activity and the consumption of antiretroviral therapy^29^. This reduction trend may be explained by earlier diagnosis and implementation of the curative intervention. The HIV screening programs, prophylaxis, and early use of antiretroviral therapy may lead to a decrease in the rate of HIV-related morbidity and mortality^28^. The possible reasons for the reduction of morbidity and mortality of HIV in Qatar maybe the regular implementation of HIV early screening programs for immigrants, especially if they stay for more than one month, as well as premarital, pre-employment, and antenatal screening. Furthermore, HIV treatment is available for all patients^29,30^. In the MENA region, Egypt has the fastest-increasing newly discovered HIV cases (25-30% annually in the past 10 years)^24^. Whereas, based on modelling results from the WHO mortality database, 2001–2018, Egypt had the lowest age-standardized death rate (ASDR) for males (0.2/100,000) and modelling results showed a positive percent change in ASDR for females (114.38% between 2001 and 2015). In 2018, the disparity in ASDR rates in males, measured using rate ratios was the lowest rate observed in Egypt^31^.

In this study, utilizing GBD data, we observed that the trend of HIV-related mortality was increasing in 15 out of 21 countries, stable in two countries, and decreasing in four countries. Similarly, according to UNAIDS data, there was an increase in HIV-related mortality in 13 out of 14 countries and a decrease in one country. The total death trend in most MENA countries was increasing. But looking at the last years of the graphs, the death rate due to AIDS has decreased in most countries. This suggests that in recent years, these countries have made efforts to implement programs proposed by WHO and UNAIDS aimed at reducing HIV incidence and mortality, although the degree of success achieved thus far may be limited^32,33^. Only in Iran, Iraq, and Libya, the death trend has always been increasing, even in the last years of the study. Similar to the results presented here, Hasankhani, et al., in separate modelling revealed an ascending trend for the incidence and mortality of this disease in Iran over the past three decades^34^. According to the results of another study carried out in Iran, the raw number of mortality, incidence, and burden of HIV increased from 2008 to 2016 in Iran^35^. Studies about the trends of HIV incidence in Iran have shown that a change in the pattern of HIV transmission from injecting the drug to unsafe sexual contact has resulted in an increased number of HIV-positive cases among women^36^. Another explanation for the higher incidence could be attributed to the shortage of educational and preventive programs, lack of knowledge, social stigma, low access to counselling and diagnostic service, and prognostic diagnostic technique in the Iranian population^34^. The increase in HIV infection notifications is difficult to interpret because of changes in the availability of tests, and the willingness of both health professionals and individuals to be tested^37^.

This study predicted incidence and mortality results based on both GBD and UNAIDS databases. Based on GBD data, both incidence and mortality rates were decreasing in Qatar, Bahrain, Kuwait, and Lebanon. However, based on UNAIDS data, it was estimated that the incidence rate will decrease in Somalia, Djibouti, and Morocco, and the mortality rate will decrease only in Qatar. Based on both databases, it was estimated that the incidence and mortality rate will increase for most of the MENA countries, with the largest increase predicted for Iran. In 2019, Khalifa et al. conducted a study to predict the number of people living with HIV and the number of new HIV infections worldwide using UNAIDS data and Spectrum software. The findings indicated that the number of people living with HIV in the MENA region would increase until 2030 and gradually decrease thereafter, with a similar trend observed for the number of new HIV infections. However, the study did not report these indices by country, providing regional-level projections instead^38^.

In this study, different epidemiological patterns are observed in the five periods identified by the Joinpoint Regression analysis during the studied period. In most countries in the MENA region, the rising tendency in the incidence and mortality rate was the most pronounced in trends 1 and 2 (1990–2004). The drop in these rates can be explained by increased access to prevention, early diagnosis, and care services for HIV infection^37^. Significant progress in HIV response in the region has been witnessed in recent years. For instance, Algeria and Morocco have made remarkable progress in growing access to HIV care services. However, other countries, including Somalia and Sudan, have encountered considerable challenges^1,39^. A notable gap between available care services and required facilities existed in the region; the existing resources in 2020 were less than 20% of what is needed to boost HIV programs and achieve the 2025 targets^1^ Progress towards the 2030 targets will depend on cooperation between organizations, linking the HIV service to efforts to reach universal health coverage and improved access to reproductive health and social protection systems. These programs will be achieved with a stronger commitment from governments and more emphasis on the recognition of the social and economic impacts of HIV on the Sustainable Development Goals^1^.

Despite the expectation of similar results, some countries showed completely different trends in the data obtained from the two different datasets (GBD and UNAIDS). For example, the death rate in Egypt and Lebanon decreased based on GBD data but increased based on UNAIDS data. Similarly, the incidence rate for Qatar was decreasing based on GBD data, but increasing based on UNAIDS data. Except for some inconsistencies mentioned in the results obtained from the two different data, the incidence and mortality rates in the rest of the countries in the two data although not the same had almost similar results. One of the reasons for this discrepancy is that the GBD data calculated age-adjusted incidence and mortality rates, while the UNAIDS data calculated incidence and mortality rates. Another reason may be the different sources of data collection in some countries, which led to different results. In general, in countries where both incidence and mortality rates are decreasing, it can be concluded as a sign of correct policy making and planning and the accurate implementation of these programs. In countries where both the incidence and mortality rates are increasing, it is probably a sign of the lack of a correct program or proper implementation of existing programs. In these countries, it is difficult to conclude that the increase in incidence is due to the improvement in disease diagnosis. In countries where one of the incidence or mortality rates is increasing and the other is decreasing, it will be a very complicated task to interpret the results and more investigations will be needed.

## Limitation

There are several limitations to the present analysis that need to be considered. First, we used two databases (GBD and UNIAIDS) to compute the AAPC. These databases can recognise only in-care patients diagnosed by physicians; therefore, there is a possibility of underestimating the results in modelling. Hence, our findings of modelling may underestimate the true incidence and mortality rate of HIV in the region. Although the GBD 2019 made various modifications to the source and evaluation of the HIV incidence to improve data accuracy, it was obvious that some deviations in the precision and completeness of the GBD data were unavoidable. To come up with this issue, we used two databases for modelling to cross-validate the results. Thus, both databases to estimate the trend of HIV infection in MENA countries have their particular advantage and restriction. The results of both of them showed a significant increase in incidence and mortality rates in most countries in the region, so there are not enough claims to identify a better database for our modelling. The outputs of the two sources supported each other in the majority of countries.

Second, it is essential to acknowledge the potential risk of the ecological fallacy, which arises from making individual-level inferences based on aggregate-level data. Despite the limitations, our study offers a comprehensive depiction of HIV status in MENA countries. This analysis provides an extensive overview of the trend of HIV infection and mortality in MENA countries.

## Conclusion

Our study found increasing incidence and mortality rates, as well as DALYs, associated with HIV in several countries across the MENA region. Future studies are needed to explore the underlying mechanisms for these epidemiological trends with potential risk factors incorporated into further analysis. To effectively address this rising trend and control the epidemic, policymakers must prioritize preventive measures in each country, including educating the pathways of transmission, informing and using the early detection methods, and timely treatment. We suggest that governments should strengthen HIV prevention and treatment programs, especially in countries with high incidence and mortality of HIV. Special attention should be directed toward improving HIV testing scale-up approaches, designing targeted interventions, expanding harm reduction services, and enhancing HIV surveillance systems. There is still plenty of room for improvement to achieve public health goals, such as HIV elimination in the MENA. Some structural interventions like addressing stigma and discrimination, and implementing supportive laws for key populations and people living with HIV are necessary to manage the epidemic in this region.

### Supplementary Information

Supplementary Tables.

## Data Availability

The datasets generated and analysed during the current study are available from the corresponding author upon reasonable request.
