# Supplementary material for: Trends and projection of incidence, mortality, and disability-adjusted life years of HIV in the Middle East and North Africa (1990–2030)
Source: Sci Rep. 2023 Aug 24;13:13859. doi: 10.1038/s41598-023-40743-z (PMC10449905; doi:10.1038/s41598-023-40743-z)
Supplement: Supplementary file 1 — Supplementary Tables. [file 41598_2023_40743_MOESM1_ESM.docx]

**Supplementary files**

Table S1. The prediction results of the incidence rate of HIV between 1993 and 2018.

| **Model** | **Latest year_Time** | **Latest year** | **APC (final segment)** | **APC% (final segment)** | **Predict** | **Predict_Time** |
| --- | --- | --- | --- | --- | --- | --- |
| 1993-2013 | 2013 | 2.130137765 | 0.9909 | 1.009909 | 2.1512453 | 2014 |
| 1994-2014 | 2014 | 2.118493422 | -3.3768 | 0.966232 | 2.046956136 | 2015 |
| 1995-2015 | 2015 | 1.900734296 | -5.0068 | 0.949932 | 1.805568331 | 2016 |
| 1996-2016 | 2016 | 1.582346957 | -13.5592 | 0.864408 | 1.367793368 | 2017 |
| 1997-2017 | 2017 | 1.602429215 | -7.4822 | 0.925178 | 1.482532256 | 2018 |
| 1998-2018 | 2018 | 1.616066658 | -6.6668 | 0.933332 | 1.508326726 | 2019 |

Table S2. The prediction evaluation results of the incidence rate of HIV between 2014 and 2019.

| **Year** | **Predicted** | **Observed** | **error ratio** | **AARD** |
| --- | --- | --- | --- | --- |
| 2014 | 2.1512453 | 2.118493422 | 0.01250791 | **0.06752818** |
| 2015 | 2.04695614 | 1.900734296 | 0.06090713 |  |
| 2016 | 1.80556833 | 1.582346957 | 0.10719701 |  |
| 2017 | 1.36779337 | 1.602429215 | 0.11160226 |  |
| 2018 | 1.48253226 | 1.616066658 | 0.06310501 |  |
| 2019 | 1.50832673 | 1.613693835 | 0.04984975 |  |

Table S3. Average Annual Percentage Change (AAPC) of age-standardized incidence rates, and incidence rates from HIV in MENA countries by gender, 1990–2019.

| **Country** | **Age-standardized incidence rates (GBD database)** | | | **Incidence rates (UNAIDS database)** | | |
| --- | --- | --- | --- | --- | --- | --- |
|  | **AAPC (95% CI)** | | | **AAPC (95% CI)** | | |
|  | **Male** | **Female** | **Both** | **Male** | **Female** | **Both** |
| Algeria | 4* (3.4, 4.7) | 3.9* (3.3, 4.6) | 4* (3.3, 4.7) | - | - | - |
| Bahrain | -3.8* (-4.2, -3.4) | -0.7 (-1.4, 0.1) | -3.3* (-4.3, -2.3) | - | - | - |
| Djibouti | 5.8* (4.6, 6.9) | 6.1* (5.4, 6.8) | 6* (5.1, 6.8) | -2.1* (-2.4, -1.7) | -1.8* (-2.2, -1.5) | -2* (-2.3, -1.6) |
| Egypt | 2.7* (2.5, 2.9) | 1.9* (1.6, 2.2) | 2.5* (2.2, 2.7) | 14* (14, 14.1) | 16* (15.9, 16.1) | 14.4* (14.3, 14.5) |
| Iran | 8.3* (7.5, 9.1) | 12.3* (11.4, 13.3) | 9.6* (8.8, 10.5) | 8.7* (8.4, 9) | 6.9* (6.6, 7.2) | 7.8* (7.2, 8.5) |
| Iraq | 5.1* (4.5, 5.6) | 5.4* (4.9, 5.9) | 5.3* (4.9, 5.7) | - | - | - |
| Jordan | 4.7* (3.8, 5.6) | 1.1* (0.2, 2) | 3.1* (2.5, 3.8) | 3.6* (3.5, 3.6) | 3.7* (3.6, 3.7) | 3.5* (3.5, 3.6) |
| Kuwait | -1.2 (-3.4, 1.2) | -1.4* (-2.6, -0.3) | -1.5 (-3.1, 0.1) | - | - | - |
| Lebanon | -1.4* (-2.1, -0.6) | 0.4 (-0.2, 1.1) | -0.8 (-1.6, 0) | 2.4* (2.3, 2.6) | -0.6* (-1.1, -0.1) | 1.9* (1.8, 1.9) |
| Libya | 4* (3.5, 4.5) | 5.8* (5.1, 6.5) | 4.8* (4.3, 5.3) | 8.8* (8.6, 9) | 9.5* (9.3, 9.7) | 8.9* (8.7, 9.1) |
| Morocco | 0.4 (-0.3, 1.1) | 2.3* (1.4, 3.1) | 1.4* (0.6, 2.2) | 0.3* (0.2, 0.4) | -2.4* (-2.5, -2.2) | -0.7* (-0.8, -0.6) |
| Oman | 5.3* (3.7, 6.9) | 5.4* (4.1, 6.6) | 5.6* (4.2, 6.9) | - | - | - |
| Palestine | 1.8* (1.6, 2.1) | 6.4* (6, 6.8) | 3.2* (2.7, 3.6) | - | - | - |
| Qatar | -5.3* (-6.2, -4.4) | -6.2* (-7.2, -5.1) | -5.6* (-6.1, -5) | 3.8* (3.1, 4.6) | 5* (4.3, 5.8) | 3.9* (3.2, 4.6) |
| Saudi Arabia | 1* (0.4, 1.6) | 2.5* (2, 3) | 1.6* (1.1, 2.1) | 7.8* (7.6, 8) | 5.9* (5.7, 6.1) | 7.3* (7.1, 7.5) |
| Somalia | -0.1 (-0.9, 0.7) | -0.2 (-0.4, 0.1) | -0.1 (-0.6, 0.3) | -4.2* (-4.5, -4) | -4.1* (-4.3, -3.9) | -4.2* (-4.4, -4) |
| Sudan | 3.4* (3.1, 3.8) | 3.4* (3.1, 3.7) | 3.4* (3.1, 3.7) | 4.5* (4.2, 4.9) | 4* (3.6, 4.4) | 4.1* (3.8, 4.5) |
| Syria | 2.4* (1.5, 3.4) | -0.8 (-4, 2.6) | 0.6 (-2.9, 4.2) | 5.5* (5.3, 5.6) | 4.5* (4.3, 4.6) | 5.2* (5.1, 5.4) |
| Tunisia | 5.3* (4.8, 5.8) | 8.2* (8, 8.5) | 6.3* (5.8, 6.8) | 6.1* (5.9, 6.2) | 9.2* (9.1, 9.4) | 7* (6.9, 7.2) |
| UAE | 5.7 (0.5, 12.3) | 5.4* (4.5, 6.3) | 5.5 (-0.6, 12) | - | - | - |
| Yemen | 1.7* (1.2, 2.2) | 3.2* (2.6, 3.9) | 2.2* (1.9, 2.5) | 3.8* (3.8, 3.8) | 3* (3, 3) | 3.5* (3.5, 3.6) |
| Total | 3.1* (1.2, 5.1) | 3.5* (3, 4) | 3.3* (2.1, 4.6) | - | - | - |
| AAPC: Average Annual Percentage Change, UAE: United Arab Emirates.  *P <0.05 versus 0 (output from joinpoint regression analysis). | | | | | | |

Table S4. Average Annual Percentage Change (AAPC) of incidence rates from HIV in MENA countries by age groups, 1990–2019.

| **Country** | **AAPC of incidence rates (GBD database)** | | | | | | **AAPC of incidence rates (UNAIDS database)** | | | | | |
| --- | --- | --- | --- | --- | --- | --- | --- | --- | --- | --- | --- | --- |
|  | **15-24** | **25-34** | **35-44** | **45-54** | **55-64** | **65-74** | **15-24** | **25-34** | **35-44** | **45-54** | **55-64** | **65-74** |
| Algeria | 3.7* (2.8, 4.6) | 1.7* (0.7, 2.7) | 3.1* (2.5, 3.7) | 4.4* (3.6, 5.2) | 7.1* (6.2, 8) | 12.6* (11.8, 13.5) | - | - | - | - | - | - |
| Bahrain | -1.1* (-1.8, -0.5) | -3.4* (-3.9, -2.9) | -4.7* (-5.3, -4.2) | -5* (-5.8, -4.3) | -0.7 (-1.5, 0.1) | 0.8 (-0.8, 2.4) | - | - | - | - | - | - |
| Djibouti | 5.5* (4.8, 6.1) | 6.1* (5.6, 6.5) | 5.9* (5.5, 6.4) | 6* (5.5, 6.4) | 6* (5.6, 6.4) | 6* (5.6, 6.4) | -3.6* (-3.9, -3.3) | -3.6* (-3.9, -3.3) | -3.6* (-3.9, -3.3) | -3.7* (-4, -3.4) | -3.6* (-3.9, -3.3) | -3.6* (-3.9, -3.3) |
| Egypt | 3.3* (2.9, 3.6) | 1.7* (1.4, 2.1) | 2.4* (2.1, 2.6) | 1.3* (1.1, 1.5) | 4* (3.3, 4.6) | 8.4* (7.7, 9.1) | 14.1* (13.9, 14.2) | 13.8* (13.7, 13.9) | 13.9* (13.8, 14.1) | 13.9* (13.7, 14) | 14.1* (14, 14.2) | 14* (13.8, 14.2) |
| Iran | 9.8* (8.7, 10.8) | 8.8* (7.8, 9.7) | 9.3* (8.3, 10.3) | 9* (8, 10.1) | 12.2* (11.6, 12.8) | 9.3* (8, 10.6) | 5.6* (5.1, 6.3) | 7.7* (7.2, 8.2) | 7.8* (7.3, 8.3) | 6.9* (6.4, 7.6) | 6.9* (6.3, 7.6) | 7.4* (6.9, 8) |
| Iraq | 6.5* (5.7, 7.3) | 3.1* (2.4, 3.7) | 4.4* (3.9, 5) | 5.8* (3.9, 7.7) | 6.6* (6, 7.1) | 8.4* (7, 9.7) | - | - | - | - | - | - |
| Jordan | 2.9* (2.1, 3.6) | 0.6* (0.1, 1.1) | 2.7* (1.7, 3.6) | 2.6* (0.9, 4.3) | 9.8* (8.4, 11.3) | 18* (16.2, 19.9) | 2.7* (2.7, 2.8) | 2.4* (2.4, 2.4) | 2.6* (2.6, 2.6) | 2.9* (2.9, 2.9) | 2.9* (2.8, 2.9) | 2.7* (2.7, 2.8) |
| Kuwait | 0.7 (-0.8, 2.3) | -2.1* (-3.7, -0.5) | -2.8* (-4.2, -1.5) | -3.8* (-5.6, -2.1) | -0.1 (-2, 1.8) | 1.3 (-4.6, 7.7) | - | - | - | - | - | - |
| Lebanon | 0.5 (-0.2, 1.2) | -2.6* (-3.2, -2.1) | -2.3* (-2.9, -1.7) | -1.9* (-3.2, -0.6) | 1.1 (-0.7, 3) | 0.7 (-0.6, 2.1) | 1.1* (1, 1.2) | 1.2* (1.1, 1.2) | 1.3* (1.3, 1.4) | 1.5* (1.5, 1.6) | 1.5* (1.5, 1.6) | 1* (1, 1.1) |
| Libya | 6.5* (5.6, 7.4) | 3.7* (2.8, 4.7) | 3.6* (3, 4.3) | 3.8* (3.1, 4.6) | 6.6* (6.2, 7) | 6.2* (4.9, 7.6) | 8.4* (8.1, 8.6) | 8.1* (7.9, 8.3) | 8* (7.8, 8.2) | 8.4* (8.1, 8.6) | 8.5* (8.3, 8.8) | 8.4* (8.2, 8.7) |
| Morocco | 2.9* (2.1, 3.7) | -0.7* (-1.3, -0.1) | -0.4 (-1.1, 0.3) | 0.5 (-0.4, 1.6) | 3.3* (2.1, 4.5) | 2.4* (1.1, 3.7) | -1.7* (-1.8, -1.6) | -1.4* (-1.5, -1.3) | -1.3* (-1.4, -1.2) | -1.1* (-1.2, -1) | -1.1* (-1.2, -1) | -0.6* (-0.8, -0.5) |
| Oman | 5.9* (3.9, 8) | 5.6* (1, 10.4) | 4.8* (2.8, 6.8) | 3.8* (1.4, 6.1) | 6.9* (4.6, 9.3) | 7.2* (4.5, 9.9) | - | - | - | - | - | - |
| Palestine | 5.7* (4.9, 6.6) | 0 (-0.4, 0.4) | 1.4* (1.1, 1.7) | 0.8* (0.3, 1.2) | 2.9* (2.5, 3.3) | 3.4* (2.3, 4.5) | - | - | - | - | - | - |
| Qatar | -4.2* (-5.1, -3.3) | -4* (-5.6, -2.3) | -8* (-8.6, -7.4) | -7.4* (-8, -6.9) | -3.9* (-5.3, -2.6) | 2.4 (-1.4, 6.3) | 3.7* (3, 4.4) | 3.6* (2.9, 4.3) | 3.2* (2.5, 4) | 2.6* (2, 3.3) | 3* (2.3, 3.7) | 3* (2.4, 3.7) |
| Saudi Arabia | 1.3* (0.8, 1.8) | 1.5* (0.9, 2.1) | 1.7* (1.2, 2.2) | 1.7 (0, 3.4) | 2.6* (2.1, 3.2) | 1.5* (0.6, 2.3) | 6.2* (6, 6.4) | 6.3* (6.1, 6.5) | 6.4* (6.2, 6.6) | 6.3* (6.2, 6.5) | 6.3* (6, 6.5) | 6.3* (6.1, 6.6) |
| Somalia | -1.4* (-1.9, -0.9) | -0.4 (-1.1, 0.4) | -0.4 (-0.8, 0) | -0.2 (-0.8, 0.4) | -0.3 (-1, 0.3) | -0.3 (-0.7, 0.1) | -5.2* (-5.5, -4.9) | -5.2* (-5.4, -4.9) | -5.3* (-5.6, -5) | -5.3* (-5.5, -5) | -5.2* (-5.5, -4.9) | -5.2* (-5.5, -4.9) |
| Sudan | 2.7* (2.3, 3.1) | 3.6* (3.2, 4) | 3.5* (3.3, 3.8) | 3.6* (3.2, 4.1) | 3.6* (3.3, 3.9) | 3.6* (3.4, 3.9) | 3.6* (3.2, 4) | 3.5* (3.1, 3.9) | 3.5* (3.1, 3.9) | 3.5* (3.2, 3.9) | 3.5* (3.1, 3.9) | 3.5* (3.2, 3.9) |
| Syria | 3.6 (-1.8, 9.2) | -2.2* (-4.2, -0.1) | -2.7 (-5.6, 0.2) | 1.2* (0.2, 2.1) | -0.4 (-3.7, 3.1) | 2.2 (-1.9, 6.5) | 3.8* (3.7, 4) | 4.1* (4, 4.3) | 4.2* (4.1, 4.4) | 4.1* (3.9, 4.2) | 4.1* (3.9, 4.2) | 4.1* (3.8, 4.3) |
| Tunisia | 9.3* (8.7, 9.8) | 2.9* (2.4, 3.4) | 5.4* (5, 5.9) | 5.1* (4.7, 5.5) | 6.2* (5.1, 7.4) | 6.8* (5.1, 8.5) | 6.8* (6.6, 7) | 6.3* (6.1, 6.4) | 5.7* (5.5, 5.8) | 6.3* (6.2, 6.5) | 6.1* (6, 6.2) | 5.7* (5.5, 5.9) |
| UAE | 6.2* (4.2, 8.2) | 3.4* (2.6, 4.1) | 2.8* (2.3, 3.4) | 3* (2.4, 3.6) | 7* (3.7, 10.4) | 16.6 (-0.1, 36.1) | - | - | - | - | - | - |
| Yemen | 3.8* (3, 4.7) | 1* (0.6, 1.5) | 0.9* (0.6, 1.2) | 1.3* (0.8, 1.9) | 4.2* (3.6, 4.9) | 3.7* (2.9, 4.5) | 2.6* (2.6, 2.6) | 2.7* (2.7, 2.7) | 3* (2.9, 3) | 2.9* (2.8, 2.9) | 2.7* (2.7, 2.7) | 3.1* (3, 3.1) |
| Total | 3.1* (2.8, 3.5) | 3.3* (2.6, 3.9) | 3.1* (2.5, 3.7) | 3.1* (2.5, 3.6) | 3.8* (1.4, 6.2) | 5* (1.4, 8.7) | - | - | - | - | - | - |
| AAPC: Average Annual Percentage Change, UAE: United Arab Emirates.  *P <0.05 versus 0 (output from joinpoint regression analysis). | | | | | | | | | | | | |

Table S5. Average Annual Percentage Change (AAPC) of age-standardized mortality rates, and mortality rates from HIV in MENA countries by gender, 1990–2019.

| **Country** | **Age-standardized mortality rates (GBD database)** | | | **Mortality rates (UNAIDS database)** | | |
| --- | --- | --- | --- | --- | --- | --- |
|  | **AAPC (95% CI)** | | | **AAPC (95% CI)** | | |
|  | **Male** | **Female** | **Both** | **Male** | **Female** | **Both** |
| Algeria | 4.1* (3.4, 4.8) | 3.5* (2.8, 4.1) | 3.8* (3.5, 4.1) | - | - | - |
| Bahrain | -2.6* (-3.6, -1.6) | -0.1 (-1.3, 1.1) | -2.5* (-3.6, -1.5) | - | - | - |
| Djibouti | 23.3* (21.5, 25) | 24.6* (23.4, 25.9) | 24.2* (23, 25.3) | 12.4* (12, 12.8) | 12.6* (12.2, 13) | 12.5* (12.1, 12.9) |
| Egypt | -2.7* (-5.1, -0.3) | -2.6* (-3.3, -1.9) | -2.7* (-4, -1.3) | 15* (14.4, 15.5) | 16.1* (15.4, 16.8) | 15.2* (14.7, 15.7) |
| Iran | 8.5* (8.1, 9) | 11.5* (11.1, 11.9) | 9.4* (8.8, 9.9) | 21* (20.8, 21.3) | 9.7* (9.6, 9.8) | 16.2* (16, 16.4) |
| Iraq | 4.4* (3.9, 4.8) | 6.5* (6.2, 6.9) | 5.7* (5.2, 6.2) | - | - | - |
| Jordan | 6.3* (5.2, 7.4) | 3.4* (2.8, 4) | 5.2* (4.6, 5.9) | 2.2* (1.5, 2.9) | 4.8* (4.3, 5.5) | 2.9* (2.1, 3.6) |
| Kuwait | -4.8* (-6.6, -3.1) | 1.1* (-1.8, 4) | -3* (-4.7, -1.4) | - | - | - |
| Lebanon | -2.1* (-2.5, -1.8) | 0.4 (-0.1, 0.9) | -1.3* (-1.8, -0.8) | 3.9* (3.7, 4.1) | 4* (3.7, 4.4) | 4.1* (3.9, 4.4) |
| Libya | 5.6* (5.3, 5.8) | 6.6* (6.3, 6.9) | 6* (5.8, 6.3) | 12.9* (12.5, 13.2) | 11.9* (11.3, 12.3) | 12.5* (12.1, 12.8) |
| Morocco | 3.9* (3.5, 4.4) | 4.2* (3.8, 4.7 | 4.1* (3.8, 4.4) | 4.4* (4.1, 4.7) | 1.9* (1.6, 2.2) | 3.5* (3.2, 3.7) |
| Oman | 7.6* (6.5, 8.7) | 7.3* (6.5, 8.2) | 7.6* (7.1, 8.1) | - | - | - |
| Palestine | 5* (4.8, 5.3) | 9.9* (9.5, 10.3) | 6.5* (6.2, 6.7) | - | - | - |
| Qatar | -3.9* (-4.4, -3.3) | -3.7* (-4.7, -2.6) | -3.8* (-4.3, -3.3) | -3.9* (-4.4, -3.3) | -2.7* (-3.3, -2) | -3.8* (-4.4, -3.1) |
| Saudi Arabia | 3.3* (3.2, 3.4) | 5.5* (5.4, 5.7) | 4.2* (4, 4.3) | 7.1* (6.7, 7.5) | 4.8* (4.3, 5.4) | 6.5* (6.1, 6.9) |
| Somalia | 14.7* (13.6, 15.8) | 15.4* (14.4, 16.4) | 15.1* (14.1, 16.1) | 5.6* (5.4, 5.8) | 4.5* (4.3, 4.7) | 5.1* (4.9, 5.3) |
| Sudan | 10* (9.2, 10.8) | 9.3* (8.8, 9.8) | 9.7* (8.8, 10.7) | 12.2* (11.6, 12.6) | 12* (11.6, 12.4) | 12.1* (11.6, 12.5) |
| Syria | 1.7* (0.6, 2.8) | 1.2* (0.1, 2.4) | 1.4* (0.3, 2.5) | 6.3* (5.8, 6.7) | 7.1* (6.8, 7.5) | 6.5* (5.9, 6.9) |
| Tunisia | 7* (6.6, 7.4) | 9.8* (9.4, 10.1) | 7.8* (7.5, 8.1) | 9.4* (9.1, 9.8) | 11* (10.6, 11.3) | 9.8* (9.6, 10) |
| UAE | 12.8* (11.8, 13.8) | 6.7* (6.3, 7) | 11.9* (10.9, 12.9) | - | - | - |
| Yemen | 1.8* (1.5, 2.1) | 2.7* (2.2, 3.1) | 2.1* (1.8, 2.4) | 3.3* (3.1, 3.5) | 2.6* (2.4, 2.8) | 3* (2.8, 3.2) |
| Total | 9.4* (8.5, 10.3) | 12.3* (11.8, 12.8) | 10.7* (9.9, 11.4) | - | - | - |
| AAPC: Average Annual Percentage Change, UAE: United Arab Emirates.  *P <0.05 versus 0 (output from joinpoint regression analysis). | | | | | | |

Table S6. Average Annual Percentage Change (AAPC) of mortality rates from HIV in MENA countries by age groups, 1990–2019.

| **Country** | **AAPC of mortality rates (GBD database)** | | | | | | **AAPC of mortality rates (UNAIDS database)** | | | | | | | |
| --- | --- | --- | --- | --- | --- | --- | --- | --- | --- | --- | --- | --- | --- | --- |
|  | **15-24** | **25-34** | **35-44** | **45-54** | **55-64** | **65-74** | **15-24** | **25-34** | **35-44** | **45-54** | **55-64** | | **65-74** | |
| Algeria | 5.5* (5.4, 5.6) | 3.2* (3.1, 3.3) | 3.4* (3.3, 3.5) | 4.6* (4.4, 4.7) | 4.6* (4.4, 4.8) | 8.2* (7.9, 8.4) | - | - | - | - | - | | - | |
| Bahrain | 1.3* (1, 1.6) | -2.8* (-3.1, -2.5) | -3.5* (-3.8, -3.2) | -2.5* (-2.7, -2.1) | -0.8* (-1.1, -0.4) | 2.1* (1.8, 2.6) | - | - | - | - | - | | - | |
| Djibouti | 21.4* (20.8, 22.2) | 21.9* (21.3, 22.7) | 25.6* (25, 26.2) | 28* (27.3, 28.6) | 27.7* (27.1, 28.3) | 26.4* (25.8, 27.2) | 15.7* (15.1, 16.2) | 10.9* (10.5, 11.2) | 17* (16.6, 17.4) | 18.8* (18.4, 19.2) | 18.5* (18.1, 19) | | 17* (16.6, 17.5) | |
| Egypt | 2.7* (2.5, 2.8) | -1* (-1.2, -0.7) | -2.5* (-2.8, -2.1) | -3.5* (-3.8, -3.1) | -4.9* (-5.3, -4.5) | -1.5* (-2, -0.9) | 13.9* (13.6, 14.4) | 13.4* (12.8, 14) | 14.5* (13.9, 15) | 14.5* (13.9, 15.1) | 14.8* (14, 15.5) | | 15.3* (14.5, 15.9) | |
| Iran | 11.6* (11.5, 11.8) | 8.3* (8.2, 8.5) | 9.2* (9.1, 9.4) | 9.1* (8.9, 9.3) | 9.1* (8.9, 9.4) | 10.6* (10.4, 10.7) | 6.3* (5.9, 6.7) | 12.3* (12, 12.5) | 17.4* (17.2, 17.6) | 17* (16.8, 17.2) | 15.7* (15.5, 15.8) | | 17.1* (16.8, 17.3) | |
| Iraq | 7.7* (7.6, 7.8) | 4.8* (4.7, 4.9) | 4* (3.9, 4.2) | 5.9* (5.8, 6.1) | 6.3* (6.2, 6.4) | 7.6* (7.4, 7.7) | - | - | - | - | - | | - | |
| Jordan | 7.5* (7.3, 7.7) | 4.1* (3.8, 4.3) | 4.8* (4.6, 5.2) | 6.1* (5.8, 6.4) | 7* (6.5, 7.4) | 14.2* (13.7, 14.7) | 3.4* (3.3, 3.6) | 2.3* (1.9, 2.9) | 2.1* (1.5, 3) | 1.2* (0.1, 2.1) | 2.8* (2.1, 3.5) | | 3.7* (3.1, 4.4) | |
| Kuwait | -1.6 (-4.1, 1) | -3.2* (-5.3, -1) | -1.5* (-3, 0) | -3* (-5.1, -0.8) | -6.1* (-8, -4.1) | -8.3* (-10.1, -6.3) | - | - | - | - | - | | - | |
| Lebanon | 1.5* (1.4, 1.6) | -1.8* (-1.9, -1.7) | -2.3* (-2.5, -2.2) | -1.5* (-1.6, -1.4) | -1.9* (-2, -1.8) | -0.5* (-0.6, -0.3) | 1.9* (1.7, 2.1) | 1.9* (1.7, 2.1) | 3.3* (3.1, 3.6) | 4.5* (4.2, 4.7) | 4.6* (4.4, 4.8) | | 5.1* (4.9, 5.4) | |
| Libya | 8.6* (8.6, 8.7) | 6.8* (6.8, 6.9) | 5.8* (5.7, 5.8) | 6.3* (6.2, 6.4) | 5.9* (5.8, 6) | 7.8* (7.6, 7.9) | 10.6* (10.2, 11) | 9.2* (8.8, 9.5) | 11.7* (11.1, 12.3) | 12.6* (12.1, 13) | 13* (12.6, 13.5) | | 13.7* (13.2, 14.2) | |
| Morocco | 6.5* (6.3, 6.6) | 4* (3.9, 4.1) | 3.8* (3.7, 3.9) | 4.6* (4.5, 4.8) | 3.6* (3.5, 3.8) | 4.9* (4.8, 5.1) | 2.7* (2.5, 2.8) | 1* (0.8, 1.3) | 2.6* (2.3, 2.8) | 3.9* (3.6, 4.1) | 4.7* (4.5, 4.9) | | 5.6* (5.4, 5.9) | |
| Oman | 7.9* (7.7, 8.1) | 6.4* (6.2, 6.7) | 7.5* (7.1, 7.9) | 8.4* (8, 8.7) | 7.5* (6.9, 8.2) | 10.7* (10.1, 11.5) | - | - | - | - | - | | - | |
| Palestine | 9.7* (9.7, 9.8) | 5.5* (5.4, 5.5) | 5.5* (5.5, 5.6) | 7.8* (7.8, 7.9) | 6.8* (6.8, 6.9) | 9.7* (9.6, 9.8) | - | - | - | - | | - | | - |
| Qatar | -0.4* (-0.6, -0.2) | -4.5* (-5.2, -3.8) | -4.9* (-5.5, -4.2) | -4.9* (-5.4, -4.2) | -2* (-2.3, -1.5) | 7.1* (6.8, 7.5) | -2.2* (-2.7, -1.6) | -5* (-5.7, -4.3) | -4.4* (-5, -3.7) | -5.2* (-5.8, -4.4) | | -5.3* (-5.9, -4.5) | | -4.2* (-4.8, -3.4) |
| Saudi Arabia | 5.2* (5.1, 5.2) | 4.2* (4.1, 4.2) | 4.4* (4.4, 4.5) | 4.6* (4.6, 4.7) | 4.6* (4.5, 4.6) | 3.9* (3.8, 4) | 5.9* (5.4, 6.5) | 4.9* (4.5, 5.3) | 5.7* (5.1, 6.3) | 5.7* (5.1, 6.4) | | 6.5* (5.9, 7.2) | | 7.4* (6.8, 8.2) |
| Somalia | 13.7* (12.9, 14.4) | 12.7* (11.8, 13.4) | 16.4* (15.5, 17.1) | 18.5* (17.6, 19.1) | 17.8* (17, 18.5) | 16.4* (15.6, 17.1) | 5.9* (5.7, 6.1) | 3.3* (3.1, 3.5) | 7.7* (7.5, 8) | 9.1* (8.9, 9.4) | | 8.4* (8.2, 8.6) | | 8.6* (8.4, 8.8) |
| Sudan | 6.8* (6.6, 6.9) | 8.4* (8.3, 8.5) | 10.5* (10.3, 10.7) | 11.3* (11.1, 11.6) | 10.9* (10.8, 11.2) | 10.1* (9.9, 10.3) | 11.9* (11.6, 12.1) | 11.6* (11.2, 12.1) | 14.3* (13.9, 14.7) | 15.5* (15, 15.9) | | 15.1* (14.7, 15.5) | | 15.4* (15, 15.8) |
| Syria | 5.2* (5, 5.3) | 2.7* (2.6, 2.8) | -0.1 (-0.4, 0.1) | 2.3* (2, 2.6) | 1.3* (1, 1.6) | 2* (1.6, 2.5) | 6.7* (6.5, 6.9) | 5.4* (5.1, 5.8) | 4.9* (4.3, 5.3) | 4.3* (3.5, 4.8) | | 4.5* (3.7, 5.2) | | 4.8* (3.9, 5.6) |
| Tunisia | 12.2* (12.1, 12.3) | 6.8* (6.7, 6.9) | 7.1* (6.9, 7.2) | 9.5* (9.4, 9.6) | 7.4* (7.3, 7.5) | 10.1* (10, 10.2) | 8.7* (8.6, 8.8) | 7.6* (7.4, 7.8) | 7.9* (7.7, 8.1) | 8.3* (8, 8.6) | | 8.8* (8.3, 9.1) | | 9.1* (8.7, 9.4) |
| UAE | 7.9* (7.8, 8.1) | 6.8* (6.7, 7) | 6.1* (6, 6.2) | 6.4* (6.3, 6.4) | 6.7* (6.3, 7.2) | 22.7* (21.8, 23.5) | - | - | - | - | | - | | - |
| Yemen | 5* (4.9, 5.1) | 2.3* (2.3, 2.4) | 1.4* (1.3, 1.5) | 1.5* (1.4, 1.6) | 1.6* (1.4, 1.7) | 3.3* (3.1, 3.4) | 3.6* (3.5, 3.7) | 2.2* (2, 2.3) | 2.4* (2.3, 2.6) | 2.5* (2.2, 2.7) | | 2.5* (2.3, 2.8) | | 3* (2.8, 3.3) |
| Total | 10.8* (10.6, 11) | 9.5* (9.3, 9.6) | 11.3* (11.1, 11.4) | 12.1* (11.9, 12.3) | 11.1* (10.9, 11.3) | 12.3* (12.1, 12.6) | - | - | - | - | | - | | - |
| AAPC: Average Annual Percentage Change, UAE: United Arab Emirates.  *P <0.05 versus 0 (output from joinpoint regression analysis). | | | | | | | | | | | | | | |

Table S7. Average Annual Percentage Change (AAPC) of age-standardized rates disability-adjusted life years from HIV in MENA countries by gender, 1990–2019.

| **Country** | **Age-** **standardized rates disability-adjusted life years (GBD database)** | | |
| --- | --- | --- | --- |
|  | **AAPC (95% CI)** | | |
|  | **Male** | **Female** | **Both** |
| Algeria | 3.5* (3.4, 3.7) | 3.1* (3, 3.3) | 3.3* (3.1, 3.4) |
| Bahrain | -2.7* (-2.9, -2.5) | -0.1 (-0.5, 0.2) | -2.5* (-2.8, -2.2) |
| Djibouti | 21.3* (20.9, 21.8) | 22.3* (21.9, 22.9) | 21.9* (21.5, 22.5) |
| Egypt | -2.3* (-2.6, -2) | -2.2* (-2.4, -2.1) | -2.3* (-2.5, -2) |
| Iran | 8.2* (8.1, 8.3) | 11.2* (11.1, 11.3) | 9.1* (9, 9.2) |
| Iraq | 5* (4.9, 5) | 6.6* (6.5, 6.6) | 5.9* (5.8, 6) |
| Jordan | 5.5* (5.1, 5.8) | 3.3* (3.1, 3.4) | 4.6* (4.4, 4.8) |
| Kuwait | -4* (-5.7, -2.2) | 1.1 (-1.8, 4.2) | -2.1* (-4.1, -0.1) |
| Lebanon | -1.8* (-1.9, -1.6) | 0.4* (0.3, 0.5) | -0.8* (-1, -0.7) |
| Libya | 5.2* (5, 5.3) | 6* (5.9, 6.1) | 5.6* (5.5, 5.7) |
| Morocco | 3.8* (3.7, 3.9) | 4.1* (4, 4.2) | 3.9* (3.8, 4) |
| Oman | 7.3* (7, 7.6) | 6.9* (6.7, 7.1) | 7.3* (7, 7.7) |
| Palestine | 5* (4.9, 5.1) | 9.4* (9.4, 9.5) | 6.4* (6.3, 6.5) |
| Qatar | -4.1* (-4.3, -3.7) | -3.9* (-4.3, -3.6) | -4* (-4.3, -3.7) |
| Saudi Arabia | 3.1* (3.1, 3.2) | 5.1* (5, 5.1) | 3.9* (3.9, 4) |
| Somalia | 13.3* (12.8, 13.7) | 13.9* (13.4, 14.3) | 13.6* (13.1, 14.1) |
| Sudan | 9.2* (8.8, 9.5) | 8.8* (8.6, 9) | 8.9* (8.7, 9.1) |
| Syria | 1.8* (1.5, 2) | 1.1* (0.8, 1.3) | 1.4* (1.1, 1.6) |
| Tunisia | 6.8* (6.7, 6.9) | 9.3* (9.2, 9.5) | 7.7* (7.6, 7.8) |
| UAE | 10* (9.8, 10.1) | 6.3* (6.3, 6.4) | 9.5* (9.3, 9.7) |
| Yemen | 2* (1.9, 2.1) | 2.8* (2.6, 2.9) | 2.2* (2.1, 2.4) |
| Total | 8.8* (8.7, 8.9) | 11.4* (11.2, 11.6) | 10.1* (9.9, 10.2) |
| APC: Average Annual Percentage Change, UAE: United Arab Emirates.  *P <0.05 versus 0 (output from joinpoint regression analysis). | | | |

Table S8. Average Annual Percentage Change (AAPC) of disability-adjusted life years rates from HIV in MENA countries by age groups, 1990–2019.

| **Country** | **AAPC of disability-adjusted life years rates (GBD database)** | | | | | |
| --- | --- | --- | --- | --- | --- | --- |
|  | **15-24** | **25-34** | **35-44** | **45-54** | **55-64** | **65-74** |
| Algeria | 5.3* (5.2, 5.5) | 3.2* (3.1, 3.3) | 3.5* (3.3, 3.6) | 4.7* (4.5, 4.8) | 4.7* (4.5, 4.9) | 8.2* (8, 8.5) |
| Bahrain | 1.2* (1, 1.5) | -2.7* (-3, -2.4) | -3.3* (-3.6, -3.1) | -2.4* (-2.7, -2.1) | -0.7* (-1, -0.3) | 2.2* (1.9, 2.6) |
| Djibouti | 19.9* (19.4, 20.5) | 20.5* (20, 21.1) | 24.4* (23.9, 25) | 26.4* (25.9, 27) | 25.9* (25.5, 26.4) | 23.9* (23.5, 24.4) |
| Egypt | 2.4* (2.3, 2.6) | -0.8* (-1.1, -0.6) | -2.2* (-2.4, -1.9) | -3.2* (-3.6, -2.9) | -4.2* (-4.5, -3.7) | -0.5 (-1, 0) |
| Iran | 10.1* (9.9, 10.2) | 7.7* (7.5, 7.8) | 8.7* (8.6, 8.8) | 8.5* (8.3, 8.6) | 8.5* (8.4, 8.6) | 9.8* (9.7, 9.9) |
| Iraq | 7.8* (7.7, 7.9) | 4.9* (4.8, 5) | 4.2* (4, 4.3) | 6* (5.9, 6.1) | 6.5* (6.3, 6.6) | 7.7* (7.5, 7.8) |
| Jordan | 7.4* (7.2, 7.6) | 4.1* (3.8, 4.3) | 4.9* (4.6, 5.2) | 6.1* (5.8, 6.4) | 7.1* (6.6, 7.5) | 14.1* (13.6, 14.7) |
| Kuwait | -1.1 (-3.1, 0.9) | -2.8* (-4.7, -0.9) | -1.4* (-2.7, 0) | -2.5* (-4.4, -0.6) | -4.8* (-6.4, -3.2) | -5.5* (-7.1, -3.9) |
| Lebanon | 1.5* (1.3, 1.6) | -1.8* (-1.9, -1.7) | -2.3* (-2.4, -2.2) | -1.5* (-1.6, -1.4) | -1.8* (-1.9, -1.7) | -0.4* (-0.5, -0.2) |
| Libya | 7.9* (7.8, 8) | 6.4* (6.4, 6.5) | 5.5* (5.4, 5.6) | 6* (6, 6.1) | 5.6* (5.6, 5.7) | 7.4* (7.3, 7.5) |
| Morocco | 5.9* (5.8, 6.1) | 3.8* (3.6, 3.9) | 3.7* (3.6, 3.8) | 4.5* (4.4, 4.7) | 3.6* (3.5, 3.8) | 4.8* (4.7, 4.9) |
| Oman | 7.7* (7.5, 7.9) | 6.4* (6.1, 6.7) | 7.5* (7.1, 7.8) | 8.4* (8, 8.8) | 7.5* (6.9, 8.2) | 10.7* (10, 11.5) |
| Palestine | 9.4* (9.3, 9.5) | 5.3* (5.3, 5.4) | 5.5* (5.4, 5.5) | 7.8* (7.7, 7.9) | 6.7* (6.7, 6.8) | 9.6* (9.5, 9.7) |
| Qatar | -0.4* (-0.6, -0.2) | -4.4* (-5.1, -3.7) | -4.8* (-5.4, -4.1) | -4.8* (-5.4, -4.1) | -1.9* (-2.3, -1.4) | 7.2* (6.8, 7.6) |
| Saudi Arabia | 4.8* (4.8, 4.9) | 4* (4, 4.1) | 4.4* (4.3, 4.4) | 4.6* (4.6, 4.7) | 4.5* (4.5, 4.6) | 3.7* (3.6, 3.8) |
| Somalia | 12.9* (12.2, 13.5) | 11.6* (10.9, 12.1) | 15.5* (14.8, 16) | 17.6* (16.9, 18.1) | 16.8* (16.1, 17.3) | 15.1* (14.5, 15.6) |
| Sudan | 6.6* (6.5, 6.8) | 8.2* (8.1, 8.4) | 10.3* (10.2, 10.6) | 11.2* (11, 11.5) | 10.8* (10.7, 11) | 10* (9.8, 10.2) |
| Syria | 5* (4.9, 5.1) | 2.7* (2.6, 2.8) | 0 (-0.3, 0.2) | 2.4* (2.2, 2.7) | 1.6* (1.3, 1.8) | 2.5* (2.1, 3) |
| Tunisia | 12* (11.9, 12) | 6.8* (6.7, 6.8) | 7* (6.9, 7.2) | 9.5* (9.4, 9.6) | 7.4* (7.3, 7.6) | 10* (9.9, 10.2) |
| UAE | 7.8* (7.7, 7.9) | 6.8* (6.6, 6.9) | 6* (5.9, 6.2) | 6.4* (6.4, 6.5) | 6.6* (6.3, 7) | 21.9* (21.2, 22.4) |
| Yemen | 4.7* (4.6, 4.8) | 2.2* (2.2, 2.3) | 1.4* (1.3, 1.5) | 1.5* (1.5, 1.6) | 1.6* (1.5, 1.7) | 3.3* (3.1, 3.4) |
| Total | 10.4* (10.2, 10.6) | 9.2* (9.1, 9.4) | 11.1* (11, 11.3) | 12* (11.9, 12.2) | 11* (10.9, 11.2) | 12* (11.7, 12.2) |
| AAPC: Average Annual Percentage Change, UAE: United Arab Emirates.  *P <0.05 versus 0 (output from joinpoint regression analysis). | | | | | | |
